# Pay gaps in the National Health Service: Gender and sexuality

**DOI:** 10.1371/journal.pone.0342384

**Published:** 2026-03-04

**Authors:** Karen Ann Mumford, Edith Aguirre, Anna Einarsdöttir, Bridget Lockyer, Melisa Sayli, Benjamin Arthur Smith

**Affiliations:** 1 School for Business and Society, University of York, York, United Kingdom; 2 IZA, Institute for the Study of Labour, Bonn, Germany; 3 ISER, University of Essex, Colchester, United Kingdom; 4 Bradford Institute for Health Research, Bradford, United Kingdom; 5 School of Economics, University of Surrey, Guildford, United Kingdom; 6 Townsville University Hospital and Health Service, Douglas, Queensland, Australia; University of Technology Sydney, AUSTRALIA

## Abstract

Studies investigating the relationship between gender, sexual identity and pay have increased in number and scope over the last three decades, enabling a greater understanding of the outcomes facing LGB+ workers in the labour market. Pay gap studies that also allow for the disclosure of sexual identity in the workplace are, however, very rare. Using a rich survey of employees from the National Health Service in England, this article considers the relationship between relative pay, LGB+ identity, and disclosure for both men and women. While the findings reveal substantial heterogeneity within the LGB + , disclosure is shown to be related to higher pay from larger returns on the endowments of LGB+ employees (men or women), especially so for men.

## Introduction

There is a large literature exploring gender stratification and related pay gaps in the social sciences [1–3]. Studies that also address potential implications of sexual orientation for pay gaps are comparatively recent and considerably rarer [4]. Using a rich survey of National Health Service (NHS) employees in England, this article seeks to provide a more complete explanation of LGB + pay gaps by including information on gender, sexual identity, coupling status, occupation, and disclosure of sexual orientation.

Explaining why there might be pay differences between LGB+ and heterosexual employees is not straight forward: the theoretical models used to predict heterosexual pay gaps provide limited insight. Becker’s commonly used Human Capital Theory (HCT) argues that wages increase with investments made in the productivity of the individual, especially investments made via formal education and on-the-job training [5–8]. While Becker went on to simultaneously hold Chairs in both Sociology and Economics at the University of Chicago, the simple linear pathway between education, productivity and pay in HCT was accepted more widely by economists [9]. As was Becker’s application of neoclassical economics’ imagery of the self-interested individual acting as a rational agent to maximise their own utility.

Neoclassical economics, whilst leveraging self-interest and rationality, considers a broad range of motivators as relevant determinants of individual behaviour [10]. Becker and his contemporaries also recognised there were many other important factors determining the relative returns to labour, including bargaining power, strategic skill and institutional rules [11]. Nevertheless, the simple relational kernel in HCT appealed to applied economists [9] and the empirical relationship between pay and education, captured in the Mincer earnings function [5–6], has proven to be a robust and relevant predictor of pay outcomes [12].

HCT rapidly evolved from an intrinsically individual perspective to a wider household context [13–14], making the model of some relevance for sociologists rather than just economists [15]. In this behavioural household context (BH-HCT), if people expect to eventually become members of a household, they may also expect to specialize in different tasks within the household to maximize the combined utility, and social capital, of the household’s members. For example, if women are expecting (or expected) to spend time out of the labour market to raise children, they may invest less in formal labour market skills and/or enter occupations that require less on-the-job training, thereby lowering their potential productivity and predicted earning capacity [13–14]. Feminists have long criticised BH-HCT, arguing it is invalid to assume women have their preferred education and career outcomes when, as girls, they were socialised to believe their primary responsibility is family care [16]. Sociologists also criticise the drivers assumed in BH-HCT, instead arguing that the segregation that men and women face in the labour market generates different education choices and career aspirations [1]. In each case, however, employers may expect that (on average) women will be less attached to the labour market and will have shorter job tenure. The outcome can become self-fulfilling if these social stereotypes are accepted, and women are denied hiring opportunities and/or training paths associated with longer tenure and higher pay [1,16–18].

It may be difficult for specific women to avoid this lower pay outcome. One way could be to engage in expensive formal education as a signal to employers that they intend to stay in the labour market to reap the returns of the investment [19]. This will be a higher risk investment for women than men if some employers simply have a “taste for discrimination”, as described in Becker’s Taste for Discrimination model [20], and deny women opportunities regardless of qualifications or if employers do not recognise the individual has different aspirations to their group average [21].

LGB+ people may be making similar decisions regarding the allocation of market and non-market work within households. They may also be facing employers practising statistical discrimination or with a “taste for discrimination” [22]. Unlike stereotyping related to physically observable characteristics (such as race or gender) though, identifying as LGB + is a potentially non-observable characteristic in the workplace if the employee does not disclose this information [23] (see however [24]).

There is a small but influential empirical literature on LGB+ versus heterosexual pay gaps based on analysis of survey respondents providing individual level information on their pay and sexual orientation [4]. Unfortunately, such studies often face a range of constraints such as having low numbers of LGB+ respondents, relying on proxy measures of sexual identity, and having limited additional explanatory variables. It is also very rare for the analyst to have information on disclosure of sexual orientation in the workplace [4, page 160]. Having information on disclosure in the workplace allows for a more complete understanding of LGB + pay gaps and, via decomposition analysis, helps to reveal differential treatment (positive or negative) of the LGB + . To the best of our knowledge, this is the first article to include direct measures of disclosure of LGB+ sexual orientation, coupling status, and occupation; thereby allowing for a more insightful interpretation of the mechanisms behind LGB + pay gaps.

The remainder of the article is structured as follows. In the next section we consider existing literature and the relationship between sexual orientation and relative wages. This is followed by: discussion of the data source and summary statistics; explanation of the methodology; and presentation of our findings. The results are further explored using decomposition analysis seeking greater insight into the determinants of the measured pay gaps. The final section presents conclusions, considers policy implications, and suggests areas for future work.

## Background and literature

In their seminal study, Badgett [25] finds that gay men or gay women (lesbians) living in same-sex relationships in the US earn less than do comparable heterosexuals living in different sex partnerships. Subsequent meta-analysis concludes that lesbians generally earn more than comparable heterosexual women, and that the negative male gay pay gap is diminishing over time [26–27]. Recent evidence suggests that the negative pay gap for gay/bisexual men (GB) in the US appears to be showing persistence at around 7 log percentage points (lpp) since 2015; while the wage advantage for lesbian/bisexual women (LB) is now close to zero [4]. These findings are arguably consistent with LGB+ employees facing pay discrimination, and also with BH-HCT if gay men are (on average) less labour market oriented than heterosexual ‘primary household-earner’ men, and lesbian women are (on average) more labour market oriented than heterosexual ‘secondary household-earner’ women [28–31].

A small pay penalty for partnered gay men relative to partnered heterosexual men is found in the UK [28]. Authors have posited that larger pay penalties for older and unpartnered gay men may be related to those men never having been legally married, leading work colleagues to infer homosexuality and, thereby, opening the door to discrimination [28]. To address this issue, without information on actual disclosure, information on same-sex legal partnership has recently been used as an indicator of how open gay men or lesbians are in their workplaces [32]. Those who have made a legal commitment to their same-sex relationship are arguably more likely to be open with their colleagues about their orientation. Findings show that, while both gay men and lesbians earn more than equivalent gender heterosexuals, same sex-legal partnerships are associated with a lower pay premium and with less promotion for gay men relative to male heterosexuals consistent with discrimination against recognisable GB status for men in UK workplaces [32], see also [29,33].

It is not possible, however, in these articles to further separate out potential discrimination effects as they do not include direct information on whether sexual orientation is identifiable in the workplace. Studies of LGB + pay gaps that include a direct measure of disclosure of sexual identity are very rare [4]: we are aware of only two. Drydakis [34] finds gay men and lesbians in Greece earn less than comparable heterosexuals (4.9 lpp less for men and 8.1 lpp for women), and those gay men and lesbians who have disclosed (are “open” about) their sexual identity in their present job earn less than those gay men and lesbian women who have not disclosed (3.1 lpp less for the men and 5.3 lpp less for the women). Plug and Berkhout [35] use data on young Dutch men, two-years post college graduation, who work full-time, and are not self-employed. They also find these gay men earn less than do comparable heterosexual men (3–4 lpp less) [35]. In contrast to Drydakis [34], however, Plug and Berkhout [35] find gay men who have disclosed receive a positive pay return of some 3–8 lpp compared to gay men who have not disclosed. They argue this pay gap is driven by well-educated disclosed gay men concentrating in lower paid, less productive, occupations where they have a competitive advantage.

Occupation is clearly a potentially important determinant of wage for LGB+ workers. Australian twins who have disclosed minority sexual status to their sibling are found to work in less prejudiced occupations, and these more tolerant occupations tend to be woman dominated [36]. Self-selection of LGB+ persons into occupations, and sectors, where they expect less prejudice, stigmatised social status (compared to heterosexuals) and discrimination may be explained by Minority Stress Theory (MST) [37]. To further avoid the stressors associated with prejudice and rejection, and the related adverse health and well-being outcomes, LGB+ workers may also hide or conceal their sexual identity in the workplace. Concealment can, however, limit access to social support, affirmation, and group-level coping mechanisms that help to protect from the negative impacts of accumulated minority group stressors [37–39].

MST has been criticised for being a deficit-based explanation [38], where the LGB+ attempt to avoid damage from stigmatism. The Minority Strength Model (MSM) argues that disclosure can actually increase the strengths of the LGB + ; raising their self-esteem, resilience and positive health outcomes in absolute terms [40–41]. While both MST and MSM come from the psychology literature and apply to health and well-being, these theories may also provide guidance when explaining labour market outcomes. School programs creating alliances between LGB + , heterosexual and cisgender students have been shown to promote social support, community connectivity and equity [41]. In a stressful group-based work environment, as health care provision often is, positive feedback may occur from disclosure with increased social support and community consciousness amongst work colleagues raising resilience and productivity for the disclosed LGB + . Analogously, as non-disclosure involves some secrecy and withdrawal from work colleagues (thereby lowering social support and community consciousness), non-disclosure could lower the productivity of those LGB+ workers (ceteris paribus).

The relationship between woman dominated occupations and low pay is well documented in the literature [42–45]. Using self-reported sexual identity survey data, within UK woman dominated occupations both lesbians and gay men are found to be more likely to possess workplace managerial authority and supervisory responsibilities than comparable heterosexuals [29]. For gay men, however, these promotions tend to be capped at lower management level, indicative of a glass ceiling (see also [46]). While these findings are consistent with Becker’s Taste for Discrimination model [20], the results may also be viewed within the Implicit Inversion Theory Framework [31]. As is argued by De Vries and Steinmetz [47], who use a combination of self-reported identity status and household composition information in Germany to show that within occupations, gender composition has little impact on GB men prospects for holding authority positions. However, working in a woman dominated occupation is associated with a higher chance of holding workplace supervisory authority for LB women [47].

While studies of relative pay including measures of sexual identity in workplace contexts are increasing in number, there are clearly many remaining important unanswered questions worth exploring [48]. Having a greater understanding of the relationship between gender, sexual identity, disclosure, and LGB + pay is important to be able to address both fairness and labour market efficiency concerns. Meta-analysis has established a strong relationship between disclosure of sexual orientation in the workplace, organisational support, and positive well-being outcomes for LGB+ employees [27,49,50].

Four related research questions are therefore addressed in this article. First, is there a pay gap between LGB+ and heterosexual workers in the sample of NHS employees in England? Second, is the interpretation of this pay gap influenced by disclosure of LGB+ status in the workplace? Third, is there a difference in the productive characteristics and/or the treatment of LGB+ employees who have disclosed or not? Fourth, are the answers to these questions different for men and women? Next, we turn to a discussion of the data and summary statistics.

## Materials and methods

### Data

To the best of our knowledge, the only data set that includes information on the determinants of pay, partnership, occupation, and disclosure of sexual orientation in the workplace is the National Health Service Employee Engagement Survey (EES-NHS) in England. The employees are all covered by the same overarching employment contract (the Agenda for Change contract) and by their own public sector pay review board (the NHS Pay Review Board, NHSPRB) [51]; which means potentially all NHS employees working in England except for doctors and dentists (doctors and dentists have a different employment contract and pay review board). The NHS is a particularly relevant workforce to survey as it is large enough to generate a suitable LGB+ sample for statistically meaningful analysis. Furthermore, the NHS employees are all working in the health sector where they share a common employer, with well recognised pay and working conditions. The NHS has a reputation for being an employer mindful of discrimination and with a diverse (in terms of nationality, ethnicity, gender, and sexual orientation) and highly unionised workforce. These commonalities help us to focus the empirical analysis presented below, however; they may also limit legitimate extrapolation of the findings outside of the NHS to other less supportive work environments in England [38,44,52,53]. This is a caveat that will be returned to in the discussion and interpretation of the results below.

The EES-NHS was launched on the 2^nd^ of January 2019 and closed on the 31^st^ of May 2019; it is a fully pre-pandemic survey. The research project, including survey, was reviewed and approved by the ELMPS (Economics, Law, Management, Politics, Sociology Departments) Ethics Committee at the University of York. Participants were recruited via a confidential online survey, distributed by the NHS. Consent was informed. All participants were provided with full information, hyperlinked to GDPR compliance pages, on the survey landing page. Participation was fully voluntary, and withdrawal could take place at any stage throughout the survey. Additional written consent was not required by the Ethics Committee.

The total sample taken from the EES-NHS includes 3724 NHS employees. The NHS Digital’s headcount data from September 2018 suggests that the potential sample frame was some 1.19 million [54], implying a response rate of less than 1% for the EES-NHS. Overall comparison of the EES-NHS with the long-running annual NHS-Staff Survey (NHS-SS), which had a response rate of 46% and some 497,00 respondents in 2018, suggests that the EES-NHS is broadly representative of the NHS workforce [55]. The EES-NHS sample has a similar gender breakdown (with around 77% women employees) and age distribution [55, Table 8]. In terms of sexual orientation, however, the EES-NHS sample has a larger proportion of respondents declaring as LGB+ (12% compared to 3.5%) and fewer respondents opting for ‘prefer not to say’ (2.3% relative to 6.5%). This greater engagement is perhaps not surprising as LGBT+ labelling was included in the advertising for the EES-NHS survey. The degree of openness about sexual orientation with all or most co-workers in the EES-NHS sample is 60.3%, and 63.5% in the National LGBT survey [56]. Similarly, 78.2% of respondents in the National LGBT Survey subsample were satisfied with their lives, and 70.9% of the EES-NHS respondents are (see [55] for more detailed comparison across these data sets).

Missing observations for variables used in the analysis below limits the usable sample from the EES-NHS to 3556 observations. As discussed above, one compensation for the overrepresentation of LGB+ employees in the EES-NHS sample is the inclusion of a reasonable number of observations in the analysis (440 LGB+). Nevertheless, the EES-NHS sample size is not large, and the sampling process was not fully random, both of which may limit the extrapolation of the findings across the NHS workforce.

### Descriptive statistics

Employees in the NHS are paid in twelve bands (or sub-bands) set by the government with advice from the NHS Pay Review Board (NHSPRB) and other parties [51]. The average hourly wage measure used below is constructed from the mid-point of the employee’s salary band, allowing for their usual working hours, and adjusting for paid overtime hours. The gender of choice from the survey respondents is used, this is clearly relevant for the 17 self-identifying transgender individuals included in the sample. Survey respondents are categorised as LGB+ according to their own choices, this is also true for the transgender respondents. Our pay gap analysis below is focused on sexual identity and includes both cis and transgender individuals. Those LGB+ employees who respond that they are open about their sexual orientation in their current job are counted as disclosed.

Variable definitions and summary statistics are presented in Table 1: pair-wise statistical testing for differences in mean values are included for the men and women samples (columns 2 and 3); LGB+ and heterosexuals (columns 4 and 5); GB + men and heterosexual men (columns 6 and 7); and LB+ women and heterosexual women (columns 8 and 9). Fuller variable definitions and further summary statistics are provided in the Supplementary Appendix, S1 Table. On average, the employees in the sample receive a salary of £16.62 per hour (column 1 of Table 1); £17.36 for men (column 2) and £16.42 for women (columns 3), suggesting a statistically significant raw gender pay gap of 4.3% or 4.4 lpp at the 95% confidence level. On average LGB+ workers receive £16.83 per hour (column 4), 1.3 lpp more that heterosexuals at £16.59 per hour (column 5), although this raw pay gap is not statistically significant at standard confidence levels.

**Table 1:** Means (and standard deviations) of variables by gender and sexual orientation.

						Men		Women	
	All	Men	Women	LGB+	HetS	GB+	HetS	LB+	HetS
	(1)	(2)	(3)	(4)	(5)	(6)	(7)	(8)	(9)
ave hourly salary £	16.62	***17.36***	***16.42***	16.83	16.59	17.71	17.23	16.03	16.46
	(6.53)	(7.27)	(6.30)	(6.80)	(6.49)	(7.61)	(7.14)	(5.86)	(6.34)
natural log ave hourly salary	2.742	***2.777***	***2.733***	2.754	2.741	2.80	2.77	2.72	2.73
	(0.36)	(0.39)	(0.35)	(0.36)	(0.36)	(0.39)	(0.39)	(0.33)	(0.36)
***LGB+ and disclose***						0.60		0.42	
***Demographics***									
man	0.21			***0.48***	***0.17***				
LGB+	0.12	***0.28***	***0.08***						
age	46.21	***45.03***	***46.53***	***41.46***	***46.88***	***41.89***	***46.24***	***41.06***	***47.01***
	(11.43)	(11.79)	(11.31)	(11.32)	(11.28)	(11.23)	(11.78)	(11.40)	(11.17)
ethnic minority	0.11	***0.14***	***0.10***	**0.09**	**0.12**	***0.10***	***0.16***	0.08	0.11
married	0.51	0.49	0.51	***0.29***	***0.54***	***0.27***	***0.57***	***0.30***	***0.53***
live in couples	0.69	0.70	0.68	***0.57***	***0.70***	***0.56***	***0.75***	***0.59***	***0.69***
dependent children	0.32	0.30	0.32	***0.14***	***0.34***	***0.07***	***0.39***	***0.21***	***0.33***
disabled	0.36	0.37	0.35	***0.45***	***0.34***	0.39	0.36	***0.50***	***0.34***
carer responsibilities	0.26	***0.19***	***0.28***	**0.23**	**0.27**	0.19	0.19	0.27	0.28
foreign born	0.12	**0.14**	**0.12**	***0.09***	***0.13***	0.11	0.15	***0.07***	***0.12***
***Qualifications***									
min qual	0.01	0.01	0.01		0.01		0.01		0.01
GCSE, D-G	0.05	0.04	0.05	***0.02***	***0.05***	0.03	0.04	**0.02**	**0.05**
GCSE, A-C	0.08	**0.07**	**0.09**	***0.05***	***0.09***	0.05	0.07	***0.05***	***0.09***
Trade	0.004	***0.01***	***0.002***	0.002	0.004		0.01	0.01	0.01
A levels	0.09	0.10	0.09	0.10	0.09	0.10	0.10	0.10	0.09
HE and TQ	0.16	0.17	0.16	0.15	0.16	0.16	0.17	0.14	0.16
first degree	0.30	0.31	0.30	0.32	0.30	0.29	0.32	0.34	0.29
higher degree	0.28	0.27	0.28	***0.32***	***0.27***	***0.34***	***0.24***	0.30	0.28
work experience	17.96	***16.02***	***18.48***	***15.12***	***18.36***	**14.92**	**16.45**	***15.29***	***18.76***
	(11.66)	(11.11)	(11.75)	(10.57)	(11.75)	(10.24)	(11.42)	(10.87)	(11.78)
***Occupation***									
allied health	0.19	0.19	0.19	0.21	0.19	0.17	0.20	***0.24***	***0.19***
ambulance (operational)	0.009	***0.03***	***0.003***	***0.04***	***0.004***	***0.06***	***0.01***	***0.03***	***0.01***
public health	0.01	**0.01**	**0.009**	0.01	0.01	0.02	0.01	0.01	0.01
commissioning manager	0.01	***0.02***	***0.01***	0.02	0.01	0.01	0.03	0.02	0.01
nurses	0.24	***0.14***	***0.27***	**0.21**	**0.25**	***0.18***	***0.12***	0.23	0.27
nursing auxiliary	0.05	0.05	0.05	0.06	0.05	0.07	0.04	0.06	0.05
social care	0.006	0.007	0.006	0.004	0.007	0.01	0.01	0.01	0.01
wider health	0.24	***0.21***	***0.25***	***0.19***	***0.25***	0.18	0.22	**0.20**	**0.25**
general management	0.09	***0.14***	***0.08***	0.11	0.09	0.15	0.14	0.07	0.08
other	0.10	***0.15***	***0.09***	0.10	0.10	***0.11***	***0.17***	0.10	0.09
health professional	0.44	***0.35***	***0.46***	0.43	0.44	**0.40**	**0.33**	0.45	0.46
***Job characteristics***									
part-time	0.24	***0.09***	***0.28***	***0.11***	***0.26***	0.06	0.10	***0.15***	***0.29***
job permanent	0.93	0.92	0.93	0.93	0.93	0.94	0.92	0.92	0.93
trade union member	0.57	***0.52***	***0.58***	0.56	0.57	0.57	0.50	0.56	0.59
current job tenure (years)	6.87	***6.20***	***7.05***	***5.53***	***7.06***	**5.48**	**6.48**	***5.58***	***7.18***
***Workplace characteristics***									
has mentor for work advice	0.47	***0.42***	***0.48***	0.45	0.47	0.43	0.42	0.48	0.48
happy with training opportunities	0.47	0.45	0.47	0.48	0.47	**0.50**	**0.43**	0.47	0.47
at least one close friend at work	0.61	***0.49***	***0.64***	***0.56***	***0.61***	0.54	0.48	**0.59**	**0.64**
has cooperative workplace	0.39	0.41	0.39	0.43	0.39	0.44	0.40	0.42	0.39
using responsive working hours	0.46	***0.39***	***0.48***	0.46	0.46	0.39	0.40	0.52	0.47
job pressure	0.55	0.55	0.56	0.55	0.56	0.50	0.56	0.58	0.55
coworker support	0.77	0.75	0.78	0.80	0.77	0.76	0.74	***0.83***	***0.77***
work-life balance	0.59	0.59	0.59	0.61	0.59	**0.64**	**0.57**	0.58	0.59
supervisor support	0.61	0.59	0.62	0.60	0.61	0.58	0.59	0.62	0.62
***NHS England Trust region***									
North of England	0.23	0.23	0.23	0.23	0.23	**0.19**	**0.24**	0.27	0.23
Midlands and East of England	0.33	***0.28***	***0.34***	***0.25***	***0.34***	**0.23**	**0.29**	***0.26***	***0.35***
London	0.15	***0.17***	***0.14***	***0.24***	***0.14***	***0.27***	***0.14***	***0.21***	***0.14***
South West	0.11	0.12	0.11	***0.07***	***0.12***	***0.07***	***0.13***	***0.06***	***0.12***
South East	0.15	***0.18***	***0.15***	***0.19***	***0.15***	**0.22**	**0.17**	0.16	0.15
***Trust type***									
Acute Specialist Trusts	0.02	***0.03***	***0.01***	***0.05***	***0.01***	0.05	0.03	***0.04***	***0.01***
Acute Trusts	0.50	0.51	0.50	***0.38***	***0.51***	***0.41***	***0.55***	***0.36***	***0.51***
Ambulance Trusts	0.01	***0.03***	***0.008***	***0.04***	***0.008***	***0.06***	***0.01***	***0.03***	***0.01***
Combined Acute and Community Trusts	0.12	0.12	0.12	0.11	0.12	0.11	0.13	0.10	0.12
Combined Mental Health/ Learning Disability and Community Trusts	0.08	**0.07**	**0.09**	***0.11***	***0.08***	***0.10***	***0.05***	0.11	0.08
Community Trusts	0.10	***0.05***	***0.11***	**0.08**	**0.10**	0.07	0.05	0.09	0.11
Mental Health/ Learning Disability Trusts	0.14	0.15	0.13	***0.20***	***0.13***	0.17	0.15	***0.23***	***0.13***
Observations	3,556	753	2,803	440	3,116	210	543	230	2,573

Mean pair differences: men (2) vs. women (3); LGB+ (3) vs. heterosexual (4); among men: GB + men (6) vs. heterosexual men (7); or among women: LB+ women (8) vs. heterosexual women (9). bold p < 0.10, bold and italic p < 0.05.

The great majority of the sample being considered are women (some 79%). Compared to the men, these women are on average older, have more work experience, are twice as likely to be nurses, half as likely to be in general management, and three times more likely to work part-time (see Table 1). The women are also more likely to have a mentor, belong to a trade union, have at least one close friend in their workplace, and make use of responsive (flexible) work hour provisions.

Sexual orientation is not evenly distributed across the genders in the sample, 28% of the men identify as GB+ (column 2 of Table 1) and 8% of the women as LB+ (column 3). Where the plus category includes asexual, demisexual, fluid, pansexual and queer respondents (see S1 Table). Amongst the GB + men, 176 are cisgender gay, 21 cisgender bisexual, 7 are cisgender plus, and 6 are transgender gay (3) or bisexual (3). Amongst the LB+ women, 109 are cisgender lesbians, 76 are cisgender bisexual, 34 are cisgender plus, and 11 transgender lesbians (9) or bisexuals (2). Disclosure of sexual orientation in the workplace is more common among men than women (60% relative to 42%, see columns 6 and 8). Considering the data in more detail, gay cisgender men are the most likely to disclose (68%) followed by cisgender lesbians (61%), and bisexual cisgender women (28%) (see Supplementary Appendix S2 Table, note disclosure for transgender LGB+ respondents refers to their sexual identity). Bisexual cisgender men are least likely to disclose (14%), however the numbers of bisexual cisgender men, transgender, and plus are all small in the sample.

Columns 6 and 7, and 8 and 9, of Table 1 reveal that the LGB+ respondents are on average younger than equivalent gender heterosexuals, they are less likely to be from an ethnic minority, married, be living with their partner, or have dependent children. They have less work experience on average, tend to have higher education (especially the men), are more likely to work as nurses if men, and less likely to work part-time if women. Workers in the NHS are located in organisational units called Trusts, there are 201 individual Trusts included in the analysis. The NHS further categorises Trusts by type according to primary function and medical specialty and by region (see S1 Table). The LGB+ respondents are more likely to work in Specialist Acute (hospital) Trusts, Ambulance Trusts and Mental Health Trusts than heterosexuals. Formal estimations of pay gaps within and between these groups are considered next.

### Estimation

Following the literature examining wage differentials developed by Becker [8] and Mincer [6], using semi-logarithmic wage equations, the earnings equation is estimated as:


$$W_{il}=X_{il}^{\prime }\beta_{l}+\varepsilon_{il},\;E\left(\varepsilon_{il}\right)=\text{0},\;l\in (a,\;b,\;p)$$ (1)

where *W*_*i*_ is the natural log of the average hourly wage, *W*, for individual *i* in group type *l*; *X*_*i*_ is a vector of explanatory variables and a constant; $\varepsilon_{il}$ is a residual term; and *a* represents comparison group a; *b* the alternative comparison group b; or *p* the pooled group of *a* and *b* combined. For example, *a* might be women, *b* might be men, and *p* would be all the men and women combined; or *a* could be set as GB + men, *b* as heterosexual men*,* and *p* would be all the men. An indicator variable identifying group membership is also included in the pooled model.

## Results and discussion

Estimating the earnings function using weighted least squares, allowing for clustering at individual Trust level throughout, the first regression specification is a parsimonious model (“Min”) including only indicator variables for gender (men) and being LGB+ (see column 1 of panel a, of Table 2). With no additional explanatory variables in the model, men earn 4.4 lpp more than women; and there is neither a sizeable nor a statistically significant pay differential between LGB+ and heterosexual employees.

**Table 2:** Determinants of log earnings (OLS).

ln(salary)	Min	Base	Broader
	(1)	(2)	(3)
**(a) Total sample**			
man	0.044***	0.040***	0.0389***
	(0.014)	(0.014)	(0.011)
LGB+	−0.0001		
	(0.0193)		
no disclose & LGB+		−0.061**	−0.049***
		(0.024)	(0.017)
disclose & LGB+		0.061**	0.044**
		(0.026)	(0.017)
Adj. R-squared	0.001	0.005	0.618
Number observations	3556		
**(b) Men sample**			
GB+	0.026		
	(0.028)		
no disclose & GB+		−0.081**	−0.025
		(0.036)	(0.028)
disclose & GB+		0.095***	0.072***
		(0.036)	(0.027)
Adj. R-squared	−0.0004	0.0122	0.573
Number observations	753		
**(c) Women sample**			
LB+	−0.018		
	(0.026)		
no disclose & LB+		−0.048	−0.050**
		(0.029)	(0.019)
disclose & LB+		0.021	0.039*
		(0.038)	(0.023)
Adj. R-squared	−0.0001	0.0002	0.637
Number observations	2803		

Standard errors in parentheses (clustered at individual Trust level). * p < 0.10, ** p < 0.05, *** p < 0.01. No disclose & sexual identity, and disclose & sexual identity, are measured relative to the omitted heterosexual category. In addition to the coefficients listed, the broader model (column 3) includes additional explanatory and control variables as shown in the complete results provided in the Supplementary Appendix Tables: S3 Table for the total sample; S4 Table for men; and S5 Table for women

In column 2 of Table 2, the LGB+ respondents are divided into those who have disclosed their sexuality identity in the workplace or not, and these two groups are compared separately to the omitted heterosexual category. Those LGB+ workers who disclose their sexual orientation earn 6.1 lpp more than heterosexuals, and those LGB + who do not disclose earn 6.1 lpp less than heterosexuals (see column 2); these two equally sized effects combined cancel out an overall LGB + pay effect (as shown in column 1).

To obtain our best measure of the relationship between disclosure and pay, it is important to control for other variables that might impact on wages. As discussed above, the Human Capital Theory (HCT) predicts wages will increase with measures related to investments made in the productivity of the individual, especially formal education, job training, and work experience [8]. In addition to these variables, the earnings function [6] estimated next is augmented with further categories of explanatory variables including: demographic variables which may affect the employee’s household setting, behavioural responses and choice of jobs (gender, LGB+ identity, having dependent children, marital status, ethnic identification, being foreign born, being disabled, being a carer, and age); occupation controls and management role; further job characteristics which are a range of variables loosely reflecting the individuals response to the labour market (working part-time, having a permanent contract, current job tenure, and being a trade union member); workplace characteristics that are associated with the workplace but can vary across employees within that work location (having an effective mentor, having supportive coworkers, having a friend in the workplace, being happy with training opportunities, being able to use responsive working hours, often feeling under pressure at work, ability to maintain work-life balance, having a supportive supervisor, and being in a cooperative work place); and Trust controls that are common to all workers in that Trust (regional location, and Trust type).

This expanded set of explanatory variables is included next in the model, this is our most augmented model specification, it is our preferred model, and we refer to it as the “broader” model. Selected results (for gender and LGB+ disclosure) from the estimation of the broader model are reported in column 3 of Table 2. (Complete results for the estimation of the broader model on the total sample are presented in column 7 of S3 Table, with selected results for pay gaps and disclosure presented in panel a of Table 2. Comparable results for men (GB+ and heterosexuals) are reported in the S4 Table column 7, and panel b of Table 2. With comparable results for women in S5 Table column 7, and panel c of Table 2.) The goodness of fit measure (adjusted R-squared) reported in the penultimate row of each panel in column 3 of Table 2 suggests the broader model is capturing a reasonable 61.8% of the total variation in earnings for the total sample, 57.3% for the men, and 63.7% for the women. There is still, however, considerable variation in workplace pay (some 40%) which is not captured by the modelling, this will be discussed further below. Comparing the results from the broader model (column 3 of Table 2) with the base model (column 2), the gender pay gap is found to be very similar at 3.9 lpp in favour of men; and decreased but still offsetting disclosure effects for LGB+ employees of −4.9 lpp without disclosure, and +4.4 lpp for those who do disclose, relative to comparable heterosexuals.

Similar results are provided for the sample of men (and women) in panel b of Table 2 (and panel c). Among the men, those GB + who disclose have a higher premium relative to comparable heterosexuals than the penalty associated with those who do not disclose (columns 2 and 3), leading to a statistically insignificant net pay gap in favor of the GB+ of 2.6 lpp (column 1). Our results differ to Plug and Berkhout [35] who found a net pay penalty for the GB among young Dutch men, although they also found a positive pay premium associated with disclosure. For women NHS employees, the positive disclosure effect is slightly outweighed by the negative non-disclosure and the overall LB + pay gap is statistically insignificant at −1.8 lpp.

The pay premiums associated with LGB+ status and disclosure reported in Table 2 are robust to the inclusion of marital status. A substantial coupling-premium is found for the total sample (see the parameter estimates for “live in couple” column 1 of S6 Table) of 0.047 lpp, 0.068 lpp for men (column 2) and 0.038 lpp for women (column 3); notably the coupling-premium is considerably higher for men than women as is well established in the literature. These findings suggest using marital status in earlier studies as a proxy for workplace disclosure of sexual orientation [32] may lead to confounded estimated relationships. It is also notable that there is not a sizable, nor a statistically significant, ethnic pay gap found in any of the models considered. Furthermore, the results are qualitatively and quantitively robust to the exclusion of transgender employees (see column 2, compared to column 1, of S7 Table).

It has been shown that bisexuals in the US are treated less favourably than other non-heterosexuals in the labour market [57–58], and that they are less likely to disclose their sexual identity accordingly. We also find that bisexuals are less likely to disclose than gay men or lesbians (see S2 Table of the Appendix). Considering pay outcomes (see panel a of S7 Table), we find the gender pay gap and the pay penalty associated with nondisclosure are qualitatively and quantitively similar for LGB+ (column 1), LG (column 3) and bisexuals (column 4). However, the positive relationship between higher pay and disclosure appears to be stronger for bisexuals: in each case (see panel a for the total sample, panel b for men, and panel c for women), the point estimate for bisexuals is more than two standard errors above the point estimate for the equivalent LGB+ or LG samples. While results for lesbians or bisexual men are not statistically significant, following the small sample approach of Bernardi et al [59] by focusing on the point estimates, the results for lesbians and bisexual men show similar patterns to those found for the other sexual identities. Conditional on small sample sizes and imprecise estimates, the analysis does not suggest bisexuals face additional pay penalties, or lower pay gains associated with disclosure, in the English NHS.

In summary, returning to the main findings reported in Table 2, a significant pay gap between LGB+ employees and comparable heterosexuals is not found, this is true in aggregate and for men and women separately. However, LGB+ workers who have disclosed their sexual identity are found to earn more relative to comparable heterosexuals, and those who have not disclosed earn less. This is also true in aggregate, and for men and women separately. Not allowing for disclosure would mask these differential outcomes for LGB+ employees in the labour market.

### Decomposing the earnings gaps

Further insight into these pay gaps can be provided via decomposition analysis [60]. Separating the pay gaps into an explained and unexplained component also helps to further address the possible confounding of productivity and discrimination (positive or negative) [35].

Following Jann [61], the approach adopted to apportion the gap in the mean earnings between groups here is discussed in Oaxaca and Ransom [62] where the reference set of estimated parameters is given by the pooled sample estimates, ${\hat{\beta }}_{p}$ (which is reported in S6 Table, column 1). The decomposition of the mean earnings gap is calculated as:


$${\overset{―}{\;W}}_{a}-{\overset{―}{W}}_{\;b}\;{=\{(\;\overset{―}{\;X}}_{a}-{\overset{―}{\;X}}_{b}){\}}^{\prime }{\hat{\beta }}_{p}+\{{(\overset{―}{\;X}}_{a}\;\prime ({\hat{\beta }}_{a}-{\hat{\beta }}_{p})+{\overset{―}{\;X}}_{b}\;\prime ({\hat{\beta }}_{p}-{\hat{\beta }}_{b})\}\;$$ (2)

where overbar denotes the mean value, for example ${\overset{―}{\;W}}_{a}=\frac{\text{1}}{n}\sum {\mathrm{\backslash nolimits}}_{i=\text{1}}^{n}W_{ia}$ for group a. The first component ${\{(\;\;\overset{―}{\;\;X}}_{a}-{\overset{―}{\;X}}_{b}{\}}^{\prime }{\hat{\beta }}_{p}$} is often referred to as the endowment (or explained component) reflecting differences in the averages of the observed characteristics across the groups; the second component $\left\{{(\overset{―}{X}}_{a}^{\prime }\left({\hat{\beta }}_{a}-{\hat{\beta }}_{p}\right)+{\overset{―}{X}}_{b}^{\prime }\left({\hat{\beta }}_{p}-{\hat{\beta }}_{b}\right)\right\}$ is the remaining portion of the gap which is usually referred to as unexplained (or sometimes as the “discrimination” component), capturing the sum of the differences in the returns to the observed characteristics of the two groups. For the men versus women decomposition, ${\hat{\beta }}_{p},$ ${\hat{\beta }}_{b}$ and ${\hat{\beta }}_{a}$ are reported in Supplementary Appendix S6 Table, columns 1, 2 and 3 respectively; and ${\overset{―}{\;X}}_{b}$ and ${\;\overset{―}{\;X}}_{a}$ are reported in S1 Table, columns 2 and 3 respectively.

Aggregate decompositions for the earnings functions are presented in Table 3, with each row summarising a separate decomposition. Beginning with the men versus women decomposition in panel a, the total gender earnings gap is 4.4 lpp in favor of men (column 1). The small, but statistically insignificant, endowment component (column 2) indicates that on average the women have more observable characteristics associated with higher pay than do the men. More than all the total pay gap is, however, associated with men receiving higher returns to their observed characteristics (as shown by the unexplained component of 5.08 lpp in column 3). We could interpret this as men receiving preferential treatment, on the characteristics contained within our dataset and the broader model specification.

**Table 3:** Decompositions of the wage gaps, broader model.

	Wage gap	Explained	Unexplained
	(1)	(2)	(3)
(a) Men versus Women	−0.04402***	0.0067	−0.0508***
	(0.0156)	(0.0112)	(0.0123)
(b) Heterosexual vs LGB+	−0.0132	0.0013	−0.01447
	(0.0195)	(0.0146)	(0.0132)
(c) Women	0.0187	0.0110	0.0077
Heterosexual vs LB+	(0.0250)	(0.0191)	(0.0155)
(d) Men	−0.0258	0.0219	−0.0477**
Heterosexual vs GB+	(0.0287)	(0.0226)	(0.0235)
(e) Non-Heterosexual LGB+	−0.1299***	−0.0285	−0.1014***
Disclosed vs Non-Disclosed	(0.0319)	(0.0242)	(0.0253)
(f) Women Non-Heterosexual LB+	−0.0687	0.0189	−0.0875**
Disclosed vs Non-Disclosed	(0.0432)	(0.0334)	(0.0379)
(g) Men Non-Heterosexual GB+	−0.1759***	−0.0684*	−0.1074***
Disclosed vs Non-Disclosed	(0.0482)	(0.0398)	(0.0353)

An insignificant pay gap is found between heterosexual and LGB+ employees (panel b), and between the heterosexual women and LB+ women (panel c). In both cases the explained and unexplained components of the decomposition are quantitatively insubstantial and statistically insignificant. Among the men (panel d), there is some evidence that the heterosexual men are receiving higher returns for their observed characteristics than the GB + men (the unexplained component is 4.77 lpp); however, the overall pay gap of 2.58 lpp between heterosexual men and GB + men is not statistically significant. It is important to note that we do not find the LB+ women have a greater endowment of observable characteristics associated with higher pay than comparable heterosexual women, nor do we find the GB + men have a lesser endowment than comparable heterosexual men, as may be predicted (ceteris paribus) by household specialization in Behavioural Human Capital Theory (BH-HCT). In both cases the explained endowment component of the decomposition is insubstantial and statistically insignificant.

In contrast, the disclosure results in panels (e) to (g) of Table 3 are striking. Those LGB + who have disclosed their sexual orientation in the workplace (row e) earn on average 12.99 lpp more than those LGB + who have not, with 78% (10.14 lpp) of this gap related to those who disclosed receiving higher returns (the unexplained component). Among the men (panel g) this result is even stronger: the GB + men who have disclosed their sexual orientation in the workplace have on average 17.59 lpp more pay than the GB + men who have not disclosed; with 6.84 lpp (39% of the gap) due to the disclosed having more of those observable characteristics associated with productivity and higher pay (the endowment component) and 10.74 lpp (61%) due to those disclosing receiving higher returns on those characteristics (the unexplained component). Among the women (panel f), those LB+ women who have disclosed also receive higher returns (unexplained component) but this is partly offset by the non-disclosing LB+ women having fewer observable characteristics associated with higher pay; the overall pay gap is smaller at 6.87 lpp and is not statistically significant. Nevertheless, in each case (panels e to g) disclosure is found to be significantly associated with sizable pay gains due to preferential (higher) rates of return for those who disclose.

In summary, LGB+ employees who have disclosed their sexual identity in English NHS workplaces are found to receive higher pay from larger rewards on their endowments (sometimes referred to as preferential treatment) than do the LGB+ employees who have not disclosed. This is true in aggregate, and for men and women. This finding is particularly strong for GB + men; GB + men who have disclosed have more observable characteristics associated with higher pay, but they also receive considerably higher returns on those characteristics, resulting in GB + men who have disclosed earning 17.59 lpp more pay than GB + men who have not disclosed.

A strength of this article stems from the EES-NHS data enabling a range of explanatory variables to be included in the analysis, helping to control for omitted variable bias and possible endogeneity. Minority Stress Theory (MST) [37–38], however, suggests LGB+ people face multiple stressors associated with stigmatized social status [1,8], leading to anxiety and adverse health outcomes. The Minority Strength Model (MSM) [40–41] suggests that with suitable supports, such as familial and legal support, LGB+ people can overcome these stressors and disclose their sexual identity. Thereby gaining access to group-level coping mechanisms, improved resilience, positive health outcomes and greater productivity. The EES-NHS does not include measures such as anxiety, resilience, health, or familial support. Being able to include a wider range of explanatory variables in the modelling may provide a fuller and more nuanced explanation of relative wages.

There are further limitations with the EES-NHS data which might reasonably be expected to influence interpretation of the findings. The results and their implications, therefore, need to be considered with caveats in mind. As discussed above, homosexuals who have disclosed their sexual identity have been found to be more likely to enter (select into) female dominated occupations and workplaces [36] where they might expect to encounter lower pay [43] but also less discrimination and fewer minority group stressors in their work environment [37–39]. We do not have a suitable explanatory variable to control for this selection effect. These LGB+ people might invest more in education and training to qualify for entry into the occupation, this would be reflected in a larger endowment component in the decomposition analysis. We do not find larger endowments for the LGB+ respondents (in aggregate or by gender) than heterosexuals (see column 2 of Table 3), however, disclosed GB + men have characteristics more closely associated with higher pay than do non-disclosed GB + men. LGB+ employees may also exert greater workplace effort in an attempt to remain in their preferred employment; this would be reflected in higher returns to their characteristics for LGB+ employees relative to heterosexuals. Higher returns (larger unexplained components) are found for disclosed LGB+ workers (in aggregate and for both genders) compared to the non-disclosed, but not for LGB+ relative to heterosexual employees (see column 3 of Table 3).

All of the employees in the sample have accepted jobs in the public health sector (the NHS), this is another possible selection effect in the analysis for which we do not have a suitable control measure. For example, it may be that (at least some) individuals who select into employment in this sector have a vocation or an intrinsic motivation to provide care [63]. These individuals may also have stronger preferences for inclusion, equity and collegial support than those found in other sectors. In aggregate, employees might be less motivated by pay, and pay gaps could be lower in the NHS accordingly [63–65].

With longitudinal data (repeated observations on the same individual over time) it is possible to control for time invariant unobserved variables such as vocation or intrinsic motivation [66]. Meta-analysis from the large literature on psychological traits suggests modest to small relationships between psychological traits, productivity, occupational choice and pay [64]. If vocation or intrinsic motivation are relatively stable over time (however see [67]), or if the relationship between intrinsic motivation and individual pay is homogenous over time, fixed-effects estimation could be used to control for the relationship between these traits and pay [64,66]. The EES-NHS survey, however, provides only a single cross section of data. Including supportive workplace measures, as in our analysis, could reasonably be expected to capture some of the impact of this potential omitted variable [64] (see also [67]). Nevertheless, the focus of the data exclusively on the NHS limits the generality of the results to employees in other sectors. Furthermore, as discussed above, while the EES-NHS sample size is large enough for statistically meaningful analysis, the survey response rate is low and the sampling process is not fully random, both of which may limit the extrapolation of the findings across the full NHS workforce.

It is also not possible with a single cross-section of data to fully address causality between disclosure and pay; individuals may have disclosed before, during or after their pay changes. In common with Plug and Berkholt [35] and Drydakis [34], we have been unable to locate a suitable identifying variable to address this concern empirically. The results show clear observational relationships between disclosure and pay, but we cannot claim to prove causality. We find that LGB+ employees who have disclosed get higher returns (or preferential treatment) on their observable characteristics than do those who have not disclosed. This unexplained component of decomposition analysis is often associated with discrimination (positive or negative) in the pay gap literature [68]. It is extremely difficult to measure discrimination, as discussed in [69] and shown by example in [70]; we seek to provide measures of pay gaps in this article. To reiterate, the goodness of fit measures presented in Table 2 suggest the broader model estimations are reasonable but are only capturing some 60% of the total variation in pay. There are clearly other factors affecting workplace pay for LGB+ that are not included in our modelling, some of which may not be possible for any data set to capture [69].

## Conclusions

Studies investigating the relationship between sexual identity and pay, that also allow for the disclosure of sexual identity are very rare; we believe this article is only the third such study [34–35]. Using a rich survey of employees from the National Health Service in England (EES-NHS) we provide a broader array of information to control for possible confounding factors; this article is the first to explore the relationship between pay, co-habitation, occupation, and disclosure of minority sexual identity to work colleagues, for both men and women.

Considering the research questions posed, a significant pay gap between LGB+ workers and comparable heterosexuals is not found, this is true in aggregate and for men and women separately. However, LGB+ employees who have disclosed their sexual identity in their workplace earn more relative to comparable heterosexuals, and those LGB+ employees who have not disclosed earn less than comparable heterosexuals. This is also true in aggregate, and for men and women separately. Not allowing for disclosure would mask these empirically offsetting pay outcomes for LGB+ employees in this labour market.

Substantial or statistically significant differences in the accumulation of observable characteristics associated with higher productivity and pay are not found to occur between LGB+ and heterosexual workers. This is true across gender, and for men and women separately. Decomposition analysis further reveals that the LGB+ employees who have disclosed their sexual identity in the workplace receive higher pay given their endowments (preferential treatment) than do the LGB+ employees who have not disclosed. This is also true in aggregate, and for men and women. This finding is particularly strong for men; those GB + men who have disclosed have observable characteristics more closely associated with higher pay and they also receive considerably higher returns on those characteristics, resulting in the GB + men who have disclosed earning 17.59 lpp more pay than the GB + men who have not disclosed.

The decomposition results reveal that the LB+ women do not have a greater endowment of the observable characteristics associated with higher pay than comparable heterosexual women, nor do the GB + men have a lesser endowment than comparable heterosexual men. In both cases the explained endowment component is insubstantial and statistically insignificant. The results also suggest that disclosing sexual minority status in the workplace is not associated, ceteris paribus, with a pay penalty. Instead, the LGB+ employees who have disclosed receive higher rates of return on their productive characteristics than do comparable undisclosed LGB + .

The survey data used in this article is of employees in the English NHS (not including doctors or dentists), a particularly relevant workforce as it is large enough to generate a suitable LGB+ sample for statistically meaningful analysis. These employees are all working in the public health sector where they share a common employer, with well recognised pay and working conditions, an employer mindful of discrimination with a diverse and highly unionised workforce. These commonalities help to focus the empirical analysis, but they limit extrapolation of the findings to other workplaces. It may be that working in the NHS attracts employees with above average inclusion and equity motivation, leading to lower pay gaps in this public health sector. It may also be that LGB+ people will engage in greater effort to work in this environment, increasing their productivity within the sector. Both of these potential sample selection effects may affect the results presented in this article, future studies using longitudinal data could address selection more effectively. Furthermore, while the sample size in the EES-NHS is reasonable for meaningful statistical analysis, the survey response rate is low and the sampling process is not fully random. This may limit the ability to extrapolate the findings within the NHS and to a broader social context, suggesting a need for additional studies. It is also not possible to identify causality between pay and disclosure with cross-sectional data such as that used in this article, the results show clear observational relationships between disclosure and pay.

Nevertheless, this article provides a valuable contribution to this under researched area of the literature, revealing substantial heterogeneity occurs within the LGB+ and that disclosure is related to higher pay from larger returns on endowments for LGB+ employees (men and women) within the NHS in England. Our results suggest workplace policies that are conducive to inclusion and increase the willingness of LGB+ employees to be open about their sexual identity with their work colleagues, may help to lessen pay gaps between heterosexuals and non-disclosing LGB + . The relationship between disclosure and higher returns to observable characteristics (endowments) established in this paper could be explained (at least in part) by the disclosed using their endowments more effectively, thereby increasing their productivity. The need to improve workforce productivity in the NHS is a pressing social concern [71]; our results suggest that including information on sexual identity and disclosure may provide insightful gains to future productivity studies. There is also clearly a need for additional studies exploring LGB + pay gaps and disclosure in other work environments. Studies exploring the determinants of the disclosure decision in the workplace would also help to contextualise the findings more fully in the future. For example, identifying how important trust is to workplace relationships and disclosure [72] or the roles of anxiety, familial support or social identity in disclosure propensity [1,6,37–40,73,74]. These future studies could help to generate a more convincing understanding of the behavioural relationships underlying theoretical explanations of the empirical findings established here [48]; and further explain the nature of the empirical relationship between inclusive psychologically safe work environments, disclosure and workforce productivity [38–40].

## Supporting information

S1 Table Definitions and means (standard deviations) of variables. (DOCX)

S2 Table Disclosure by sexual identity. (DOCX)

S3 Table Determinants of log earnings (OLS estimates), total sample. (DOCX)

S4 Table Determinants of log earnings (OLS estimates), men. (DOCX)

S5 Table Determinants of log earnings (OLS estimates), women. (DOCX)

S6 Table Determinants of log earnings, decomposition (OLS estimates). (DOCX)

S7 Table Broader model comparisons, (OLS estimates). (DOCX)

## References

[1] Reskin BF, Bielby DD. A sociological perspective on gender and career outcomes. Journal of Economic Perspectives. 2005;19(1):71–86. doi: 10.1257/0895330053148010

[2] Blau FD, Kahn LM. The gender wage gap: Extent, trends, and explanations. Journal of Economic Literature. 2017;55(3):789–865. doi: 10.1257/jel.20160995

[3] Goldin C, Kerr SP, Olivetti C. When the kids grow up: women’s employment and earnings across the family lifecycle. VOX Eu CEPR. 2022. https://cepr.org/voxeu/columns/when-kids-grow-womens-employment-and-earnings-across-family-lifecycle

[4] Badgett MVL, Carpenter CS, Sasone D. LGBTQ Economics. Journal of Economic Perspectives. 2021;35:141–71.

[5] Mincer J. Investment in human capital and personal income distribution. Journal of Political Economy. 1958;66(4):281–302. doi: 10.1086/258055

[6] Mincer J. Schooling, experience and earnings. New York: NBER. 1974.

[7] Becker G. Human capital. Columbia University Press, New York; 1964.

[8] Becker G. Human Capital. 2 ed. Chicago: University of Chicago Press. 1975.

[9] Rees A. Review of human capital: a theoretical and empirical analysis with special reference to education. Am Econ Rev. 1965;55:958–60.

[10] Marshall A. Principles of economics. London: Macmillan and Co. 1890.

[11] Reder MW, Becker G. Gary Becker’s human capital: A review article. The Journal of Human Resources. 1967;2(1):97. doi: 10.2307/144593

[12] Polachek SW. Earnings over the life cycle: The mincer earnings function and its applications. FNT in Microeconomics. 2008;4(3):165–272. doi: 10.1561/0700000018

[13] Becker G. Treatise on the Family. Cambridge, MA: Harvard University Press. 1981.

[14] Becker GS. Human Capital, Effort, and the Sexual Division of Labor. Journal of Labor Economics. 1985;3(1, Part 2):S33–58. doi: 10.1086/298075

[15] Coleman JS. The Impact of Gary Becker’s Work on Sociology. Acta Sociologica. 1993;36(3):169–78. doi: 10.1177/000169939303600302

[16] Ferber M. The study of economics: A feminist critique. AEA Paper and Proceedings. 1995;85:357–61.

[17] Aigner DJ, Cain GG. Statistical theories of discrimination in labor markets. Industrial and Labor Relations Review. 1977;30(2):175. doi: 10.2307/2522871

[18] Tilcsik A. Statistical discrimination and the rationalization of stereotypes. Am Sociol Rev. 2020;86(1):93–122. doi: 10.1177/0003122420969399

[19] Spence M. Job Market Signaling. The Quarterly Journal of Economics. 1973;87(3):355. doi: 10.2307/1882010

[20] Becker G. The economics of discrimination. Chicago: University of Chicago Press. 1957.

[21] Phelps E. The statistical theory of racism and sexism. The American Economic Review. 1972;62:659–61.

[22] Badgett MVL. Employment and sexual orientation: disclosure and discrimination in the workplace. Ellis AL, Riggle EDB. Sexual identity on the job: Issues and services. Harrington Park Press/Haworth Press. 1996. 29–52.

[23] Stenger S, Roulet TJ. Pride against prejudice? the stakes of concealment and disclosure of a stigmatized identity for Gay and Lesbian Auditors. Work, Employment and Society. 2017;32(2):257–73. doi: 10.1177/0950017016682459

[24] Einarsdóttir A, Hoel H, Lewis D. Fitting the bill? (Dis)embodied disclosure of sexual identities in the workplace. Work, Employment and Society. 2015;30(3):489–505. doi: 10.1177/0950017014568136

[25] Badgett MVL. The wage effects of sexual orientation discrimination. Industrial and Labor Relations Review. 1995;48(4):726. doi: 10.2307/2524353

[26] Klawitter M. Meta‐analysis of the effects of sexual orientation on earnings. Industrial Relations. 2014;54(1):4–32. doi: 10.1111/irel.12075

[27] Drydakis N. Sexual orientation and earnings: A meta-analysis 2012–2020. J Popul Econ. 2021;35(2):409–40. doi: 10.1007/s00148-021-00862-1

[28] Aksoy CG, Carpenter CS, Frank J. Sexual orientation and earnings: New evidence from the United Kingdom. ILR Review. 2017;71(1):242–72. doi: 10.1177/0019793916687759

[29] Aksoy CG, Carpenter CS, Frank J, Huffman ML. Gay glass ceilings: Sexual orientation and workplace authority in the UK. Journal of Economic Behavior & Organization. 2019;159:167–80. doi: 10.1016/j.jebo.2019.01.013

[30] Jepsen C, Jepsen L. Convergence over time or not? U.S. wages by sexual orientation, 2001-2018. 2020. https://repec.iza.org/dp13495.pdf

[31] Kite ME, Deaux K. Gender Belief Systems: Homosexuality and the Implicit Inversion Theory. Psychology of Women Quarterly. 1987;11(1):83–96. doi: 10.1111/j.1471-6402.1987.tb00776.x

[32] Bridges S, Mann S. Sexual Orientation, Legal Partnerships and Wages in Britain. Work, Employment and Society. 2019;33(6):1020–38. doi: 10.1177/0950017019873265

[33] Arabsheibani GR, Marin A, Wadsworth J. Gay Pay in the UK. Economica. 2005;72(286):333–47. doi: 10.1111/j.0013-0427.2005.00417.x

[34] Drydakis N. Effect of Sexual Orientation on Job Satisfaction: Evidence from Greece. Industrial Relations. 2014;54(1):162–87. doi: 10.1111/irel.12080

[35] Plug E, Berkholt P. Sexual orientation, disclosure and earnings. 3290. IZA. 2008. https://www.iza.org/publications/dp/3290/sexual-orientation-disclosure-and-earnings

[36] Meyer IH. Prejudice, social stress, and mental health in lesbian, gay, and bisexual populations: Conceptual issues and research evidence. Psychol Bull. 2003;129(5):674–97. doi: 10.1037/0033-2909.129.5.674 12956539 PMC2072932

[37] Meyer IH. Resilience in the study of minority stress and health of sexual and gender minorities. Psychology of Sexual Orientation and Gender Diversity. 2015;2(3):209–13. doi: 10.1037/sgd0000132

[38] Frost DM, Meyer IH. Minority stress theory: Application, critique, and continued relevance. Curr Opin Psychol. 2023;51:101579. doi: 10.1016/j.copsyc.2023.101579 37270877 PMC10712335

[39] Swann WB. Self-Verification Theory. Handbook of Theories of Social Psychology. SAGE Publications Ltd. 23–42. doi: 10.4135/9781446249222.n27

[40] Perrin PB, Sutter ME, Trujillo MA, Henry RS, Pugh M Jr. The minority strengths model: Development and initial path analytic validation in racially/ethnically diverse LGBTQ individuals. J Clin Psychol. 2020;76(1):118–36. doi: 10.1002/jclp.22850 31468539 PMC6908758

[41] Plug E, Webbink D, Martin N. Sexual Orientation, Prejudice, and Segregation. Journal of Labor Economics. 2014;32(1):123–59. doi: 10.1086/673315

[42] Antecol H, Jong A, Steinberger M. The sexual orientation wage gap: The role of occupational sorting and human capital. ILR Review. 2008;61(4):518–43. doi: 10.1177/001979390806100405

[43] Mumford K, Smith PN. What determines the part-time and gender earnings gaps in Britain: Evidence from the workplace. Oxford Economic Papers. 2008;61(Supplement 1):i56–75. doi: 10.1093/oep/gpn041

[44] Ahmed AM, Alden L, Hammarstedt M. Sexual orientation and occupational rank. Economics Bulletin. 2011;31:2422–33.

[45] Finnigan R. Rainbow-collar jobs? Occupational segregation by sexual orientation in the United States. Socius: Sociological Research for a Dynamic World. 2020;6. doi: 10.1177/2378023120954795

[46] Frank J. Gay Glass Ceilings. Economica. 2006;73(291):485–508. doi: 10.1111/j.1468-0335.2006.00516.x

[47] de Vries L, Steinmetz S. Sexual orientation, workplace authority and occupational segregation: Evidence from Germany. Work, Employment and Society. 2023;38(3):852–70. doi: 10.1177/09500170231158513

[48] Merton RK. The Bearing of Empirical Research upon the Development of Social Theory. American Sociological Review. 1948;13(5):505. doi: 10.2307/2087142

[49] Wax A, Coletti KK, Ogaz JW. The benefit of full disclosure: A meta-analysis of the implications of coming out at work. Organizational Psychology Review. 2018;8:3–30.

[50] OECD. Over the rainbow? The road to LGBTI inclusion. OECD Publishing, Paris; 2020. 10.1787/8d2df1a8-en

[51] OME Office for Manpower Economics. Advisory web pages. https://www.gov.uk/government/organisations/nhs-pay-review-body

[52] Bryson A. Pay equity after the Equality Act 2010: does sexual orientation still matter?. Work, Employment and Society. 2016;31(3):483–500. doi: 10.1177/0950017016664678

[53] Wang J, Gunderson M, Wicks D. The earnings effect of sexual orientation: British evidence from worker‐firm matched data. Brit J Industrial Rel. 2018;56(4):744–69. doi: 10.1111/bjir.12304

[54] NHS England. NHS Workforce Statistics. 2018. https://digital.nhs.uk/data-and-information/publications/statistical/nhs-workforce-statistics/september-2018

[55] Einarsdóttir A, Mumford K, Birks Y, Aguirre E, Lockyer B, Sayli M. LGBT employee networks within the NHS: Technical report and data addendum. York: University of York. 2020. https://www.researchgate.net/publication/344670897

[56] Government Equalities Office GEO. National LGBT Survey Research Report. Manchester: Government Equalities Office. https://assets.publishing.service.gov.uk/media/5b3b2d1eed915d33e245fbe3/LGBT-survey-research-report.pdf

[57] Mize TD. Sexual Orientation in the Labor Market. Am Sociol Rev. 2016;81(6):1132–60. doi: 10.1177/0003122416674025

[58] Liu X, Cai S, Glasser A, Volberg T, Polansky JR, Fauss DJ, et al. Effect of H-7 on cultured human trabecular meshwork cells. Mol Vis. 2001;7:145–53. 11436001

[59] Bernardi F, Chekhaia L, Leopold L. Sing me a song with social significance: The (mis)use of statistical significance testing in European sociological research. European Sociological Review. 2017;33:1–16.

[60] Oaxaca R. Male-Female Wage Differentials in Urban Labor Markets. International Economic Review. 1973;14(3):693. doi: 10.2307/2525981

[61] Jann B. The Blinder–Oaxaca Decomposition for Linear Regression Models. The Stata Journal: Promoting communications on statistics and Stata. 2008;8(4):453–79. doi: 10.1177/1536867x0800800401

[62] Oaxaca RL, Ransom MR. On discrimination and the decomposition of wage differentials. Journal of Econometrics. 1994;61(1):5–21. doi: 10.1016/0304-4076(94)90074-4

[63] Heyes A. The economics of vocation or “why is a badly paid nurse a good nurse”?. J Health Econ. 2005;24(3):561–9. doi: 10.1016/j.jhealeco.2004.09.002 15811543

[64] Vella M. The relationship between the Big Five personality traits and earnings: Evidence from a meta‐analysis. Bulletin of Econ Res. 2024;76(3):685–712. doi: 10.1111/boer.12437

[65] Bleidorn W, Hill PL, Back MD, Denissen JJA, Hennecke M, Hopwood CJ, et al. The policy relevance of personality traits. Am Psychol. 2019;74(9):1056–67. doi: 10.1037/amp0000503 31829685

[66] Amoroso S, Magazzini L, Bruno R. The identification of time-invariant variables in panel data model: exploring the role of science in firms’ productivity. IZA Institute of Labor Economics. 2022. https://docs.iza.org/dp15708.pdf

[67] La Nauze A. Sexual orientation–based wage gaps in Australia: The potential role of discrimination and personality. The Economic and Labour Relations Review. 2015;26(1):60–81. doi: 10.1177/1035304615570806

[68] van der Meulen Rodgers Y. A primer of wage gap decompositions in the analysis of labor market discrimination. Rodgers W. Handbook of the Economics of Discrimination. Cheltenham UK: Edward Elgar. 2006.

[69] Badgett MVL. Discrimination based on sexual orientation: a review of the literature in economics and beyond. Rodgers W. Handbook of the Economics of Discrimination. Cheltenham UK: Edward Elgar. 2006.

[70] Goldin C, Rouse C. Orchestrating Impartiality: The Impact of “Blind” Auditions on Female Musicians. American Economic Review. 2000;90(4):715–41. doi: 10.1257/aer.90.4.715

[71] Moody N, Powell T. NHS Productivity. House of Commons Library. 2025. https://commonslibrary.parliament.uk/research-briefings/cbp-10313/

[72] Zac P. The neuroscience of trust. Harvard Business Review. 2017;:84–90.

[73] Tajfel H, Turner J. An integrative theory of intergroup conflict. Austin W, Worchel S. The Social Psychology of Intergroup Relations. Monterey, CA: Wadsworth. 1979.

[74] Akerlof GA, Kranton RE. Economics and Identity*. Quarterly Journal of Economics. 2000;115(3):715–53. doi: 10.1162/003355300554881

