# Supplementary material for: Pay gaps in the National Health Service: Gender and sexuality
Source: PLoS One. 2026 Mar 4;21(3):e0342384. doi: 10.1371/journal.pone.0342384 (PMC12959664; doi:10.1371/journal.pone.0342384)
Supplement: S1 Table — (DOCX) [file pone.0342384.s001.docx]

| **S1 TABLE. Definitions and means (standard deviations) of variables.** | | | | | | |
| --- | --- | --- | --- | --- | --- | --- |
|  | **Means (standard deviations)** | | | | | **Definitions** |
|  | **Total** | **Men** | **Women** | **LGB+** | **Heterosexual** |  |
|  | (1) | (2) | (3) | (4) | (5) |  |
|  |  |  |  |  |  |  |
| salary | 16.62 | ***17.36*** | ***16.42*** | 16.83 | 16.59 | Average hourly pay in GBP (full time equivalent). |
|  | (6.53) | (7.27) | (6.30) | (6.80) | (6.49) |  |
| natural log salary | 2.742 | ***2.777*** | ***2.733*** | 2.754 | 2.741 |  |
|  | (0.36) | (0.39) | (0.35) | (0.36) | (0.36) |  |
| ***Sexuality*** |  |  |  |  |  |  |
| LGB+ | 0.12 | ***0.28*** | ***0.08*** |  |  | Respondent LGB+ |
| disclose | 0.06 | ***0.17*** | ***0.03*** | 0.51 |  | Open about sexuality at workplace |
|  |  |  |  |  |  |  |
| ***Demographics*** |  |  |  |  |  |  |
| male | 0.21 |  |  | ***0.48*** | ***0.17*** | Respondent is male |
| age | 46.21 | ***45.03*** | ***46.53*** | ***41.46*** | ***46.88*** | Age of respondent |
|  | (11.43) | (11.79) | (11.31) | (11.32) | (11.28) |  |
| ethnic minority | 0.11 | ***0.14*** | ***0.10*** | **0.09** | **0.12** | Ethnic group not white (Mixed; Asian; Black/Black British; Arab or Other) |
| married | 0.51 | 0.49 | 0.51 | ***0.29*** | ***0.54*** | Married |
| lives in couple | 0.69 | 0.70 | 0.68 | ***0.57*** | ***0.70*** | Living together |
| dependent children | 0.32 | 0.30 | 0.32 | ***0.14*** | ***0.34*** | Has dependent children |
| disability | 0.36 | 0.37 | 0.35 | ***0.45*** | ***0.34*** | Long-standing illness, health problem or disability |
| carer | 0.26 | ***0.19*** | ***0.28*** | **0.23** | **0.27** | Look after or give support to family/friend due to health or old age |
| foreign born | 0.12 | **0.14** | **0.12** | ***0.09*** | ***0.13*** | Born outside UK |
|  |  |  |  |  |  |  |
| ***Qualifications*** |  |  |  |  |  |  |
| min qual | 0.01 | 0.01 | 0.01 |  | 0.01 | Below O level or no recognised qualifications |
| GCSE D-G | 0.05 | 0.04 | 0.05 | ***0.02*** | ***0.05*** | O level or GCSE grades D-G (national exams usually taken at age 16). |
| GCSE A-C | 0.08 | **0.07** | **0.09** | ***0.05*** | ***0.09*** | O level or GCSE grades A-C (national exams usually taken at age 16). |
| trade | 0.004 | ***0.01*** | ***0.002*** | 0.002 | 0.004 | Trade apprenticeships |
| A levels | 0.09 | 0.10 | 0.09 | 0.10 | 0.09 | A levels and others (national exams usually taken at age 18 at end of secondary education). |
| HE and TQ | 0.16 | 0.17 | 0.16 | 0.15 | 0.16 | Diploma in higher education, teaching qualifications and others |
| first degree | 0.30 | 0.31 | 0.30 | 0.32 | 0.30 | First degree and PGCE |
| higher degree | 0.28 | 0.27 | 0.28 | ***0.32*** | ***0.27*** | Higher degree or postgraduate |
|  |  |  |  |  |  |  |
| work experience | 17.96 | ***16.02*** | ***18.48*** | ***15.12*** | ***18.36*** | Years of potential work experience |
|  | (11.66) | (11.11) | (11.75) | (10.57) | (11.75) |  |
|  |  |  |  |  |  |  |
| ***Occupation*** |  |  |  |  |  |  |
| allied health | 0.19 | 0.19 | 0.19 | 0.21 | 0.19 | Allied health professional, healthcare scientist, scientific and technical |
| ambulance (operational) | 0.009 | ***0.03*** | ***0.003*** | ***0.04*** | ***0.004*** | Ambulance (operational) |
| public health | 0.01 | **0.01** | **0.009** | 0.01 | 0.01 | Public health/health improvement |
| commissioning manager | 0.01 | ***0.02*** | ***0.01*** | 0.02 | 0.01 | Commissioning manager/support staff |
| nurses | 0.24 | ***0.14*** | ***0.27*** | **0.21** | **0.25** | Registered nurse and midwives |
| nursing auxiliary | 0.05 | 0.05 | 0.05 | 0.06 | 0.05 | Nursing auxiliary, nursing assistant, health care assistants |
| social care | 0.006 | 0.007 | 0.006 | 0.004 | 0.007 | Social care |
| wider health | 0.24 | ***0.21*** | ***0.25*** | ***0.19*** | ***0.25*** | Wider healthcare team |
| general management | 0.09 | ***0.14*** | ***0.08*** | 0.11 | 0.09 | General management |
| other | 0.10 | ***0.15*** | ***0.09*** | 0.10 | 0.10 | Other |
|  |  |  |  |  |  |  |
| health professional | 0.44 | ***0.35*** | ***0.46*** | 0.43 | 0.44 | Respondent is health professional |
|  |  |  |  |  |  |  |
| ***Job characteristics*** |  |  |  |  |  |  |
| part time | 0.24 | ***0.09*** | ***0.28*** | ***0.11*** | ***0.26*** | Part-time work |
| job permanent | 0.93 | 0.92 | 0.93 | 0.93 | 0.93 | Has a permanent contract |
| trade union member | 0.57 | ***0.52*** | ***0.58*** | 0.56 | 0.57 | Member of a trade union |
| current job tenure (years) | 6.87 | ***6.20*** | ***7.05*** | ***5.53*** | ***7.06*** | Years in current job |
|  |  |  |  |  |  |  |
| ***Workplace characteristics*** |  |  |  |  |  |  |
| has mentor for work advice | 0.47 | ***0.42*** | ***0.48*** | 0.45 | 0.47 | Has mentor/coach for work advice |
| happy with training opportunities | 0.47 | 0.45 | 0.47 | 0.48 | 0.47 | Satisfied with opportunities to develop skills |
| at least one close work friend | 0.61 | ***0.49*** | ***0.64*** | ***0.56*** | ***0.61*** | At least one close friend in workplace |
| cooperative workplace | 0.39 | 0.41 | 0.39 | 0.43 | 0.39 | Feel workplace is cooperative |
| uses responsive work hours | 0.46 | ***0.39*** | ***0.48*** | 0.46 | 0.46 | Using at least one of the responsive work hours (flexi-time, reduced hours, same hours fewer days and paid leave to care) |
| job pressure | 0.55 | 0.55 | 0.56 | 0.55 | 0.56 | Job makes feel pressure always and often |
| coworker support | 0.77 | 0.75 | 0.78 | 0.80 | 0.77 | Has supportive colleagues |
| work-life balance | 0.59 | 0.59 | 0.59 | 0.61 | 0.59 | Maintains work-life balance |
| supervisor support | 0.61 | 0.59 | 0.62 | 0.60 | 0.61 | Supervisor responds to suggestions |
|  |  |  |  |  |  |  |
|  |  |  |  |  |  |  |
| ***NHS England region*** |  |  |  |  |  |  |
| North of England | 0.23 | 0.23 | 0.23 | 0.23 | 0.23 |  |
| Midlands and East of  England | 0.33 | ***0.28*** | ***0.34*** | ***0.25*** | ***0.34*** |  |
| London | 0.15 | ***0.17*** | ***0.14*** | ***0.24*** | ***0.14*** |  |
| South West | 0.11 | 0.12 | 0.11 | ***0.07*** | ***0.12*** |  |
| South East | 0.15 | ***0.18*** | ***0.15*** | ***0.19*** | ***0.15*** |  |
|  |  |  |  |  |  |  |
| ***Trust type*** |  |  |  |  |  |  |
| Acute Specialist Trusts | 0.02 | ***0.03*** | ***0.01*** | ***0.05*** | ***0.01*** |  |
| Acute Trusts | 0.50 | 0.51 | 0.50 | ***0.38*** | ***0.51*** |  |
| Ambulance Trusts | 0.01 | ***0.03*** | ***0.008*** | ***0.04*** | ***0.008*** |  |
| Combined Acute and Community Trusts | 0.12 | 0.12 | 0.12 | 0.11 | 0.12 |  |
| Combined Mental Health / Learning Disability and Community Trusts | 0.08 | **0.07** | **0.09** | ***0.11*** | ***0.08*** |  |
| Community Trusts | 0.10 | ***0.05*** | ***0.11*** | **0.08** | **0.10** |  |
| Mental Health / Learning Disability Trusts | 0.14 | 0.15 | 0.13 | ***0.20*** | ***0.13*** |  |
|  |  |  |  |  |  |  |
| Observations | 3,556 | 753 | 2,803 | 440 | 3,116 |  |
| Mean pair differences: men (2) vs. women (3); LGB+ (3) vs. heterosexual (4), bold p<0.10, bold and italic p<0.05. | | | | | | |
