# Supplementary material for: Pay gaps in the National Health Service: Gender and sexuality
Source: PLoS One. 2026 Mar 4;21(3):e0342384. doi: 10.1371/journal.pone.0342384 (PMC12959664; doi:10.1371/journal.pone.0342384)
Supplement: S2 Table — (DOCX) [file pone.0342384.s002.docx]

| **S2 TABLE. Disclosure by sexual identity.** | | | | | | | | | | | |
| --- | --- | --- | --- | --- | --- | --- | --- | --- | --- | --- | --- |
| Sexual identity | **Men** | | | | Total | **Women** | | | | Total | |
|  | Disclosure | % | Non-disclosure | % |  | Disclosure | % | Non-disclosure | % |  |  |
| Gay | 119 | 67.6 | 57 | 32.4 | 176 | - | - | - | - | - |  |
| Lesbian | - | - | - | - | - | 66 | 60.6 | 43 | 39.4 | 109 |  |
| Bisexual | 3 | 14.3 | 18 | 85.7 | 21 | 21 | 27.6 | 55 | 72.4 | 76 |  |
| Plus | 2 | 28.6 | 5 | 71.4 | 7 | 6 | 17.6 | 28 | 82.4 | 34 |  |
| LGB+ Transgender | 3 | 50.0 | 3 | 50.0 | 6 | 4 | 36.4 | 7 | 63.6 | 11 |  |
| Total | 127 | 60.5 | 83 | 39.5 | 210 | 97 | 42.2 | 133 | 57.8 | 230 |  |
