# Supplementary material for: Pay gaps in the National Health Service: Gender and sexuality
Source: PLoS One. 2026 Mar 4;21(3):e0342384. doi: 10.1371/journal.pone.0342384 (PMC12959664; doi:10.1371/journal.pone.0342384)
Supplement: S3 Table — (DOCX) [file pone.0342384.s003.docx]

| **S3 TABLE. Determinants of log earnings (OLS estimates), total sample.** | | | | | | | | | |
| --- | --- | --- | --- | --- | --- | --- | --- | --- | --- |
| Dependent variable is ln(salary) | (1) | (2) | (3) | (4) | (5) | (6) | (7) | (8) | (9) |
|  | Min | Min+ | Base | +HC | +Demog | +Occup | +Job/Work | Broader | Coupled |
| male | 0.0440*** | 0.0440*** | 0.0396*** | 0.0660*** | 0.0624*** | 0.0500*** | 0.0423*** | 0.0388*** | 0.0509*** |
|  | (0.0139) | (0.0140) | (0.0137) | (0.0121) | (0.0118) | (0.0106) | (0.0111) | (0.0109) | (0.0134) |
| LGB+ |  | -0.0001 |  |  |  |  |  |  |  |
|  |  | (0.0193) |  |  |  |  |  |  |  |
| no disclose&LGB+ |  |  | -0.0612** | -0.0558*** | -0.0323* | -0.0352** | -0.0411** | -0.0493*** | -0.0495** |
|  |  |  | (0.0238) | (0.0175) | (0.0171) | (0.0157) | (0.0159) | (0.0165) | (0.0232) |
| disclose & LGB+ |  |  | 0.0614** | 0.0330* | 0.0572*** | 0.0577*** | 0.0557*** | 0.0435** | 0.0364 |
|  |  |  | (0.0263) | (0.0193) | (0.0187) | (0.0178) | (0.0180) | (0.0172) | (0.0253) |
|  | | |  |  |  |  |  |  |  |
| Qualifications (omitted group: min qual) | | |  |  |  |  |  |  |  |
| O level |  |  |  | 0.0738 | 0.0865** | 0.0087 | 0.0030 | 0.0001 | 0.0501 |
|  |  |  |  | (0.0488) | (0.0437) | (0.0506) | (0.0507) | (0.0517) | (0.0612) |
| GCSE |  |  |  | 0.1440*** | 0.1505*** | 0.0424 | 0.0362 | 0.0275 | 0.0892 |
|  |  |  |  | (0.0537) | (0.0488) | (0.0522) | (0.0521) | (0.0531) | (0.0679) |
| trade |  |  |  | 0.2148** | 0.2165** | 0.1102 | 0.0920 | 0.0938 | 0.1905* |
|  |  |  |  | (0.0962) | (0.0962) | (0.0915) | (0.0787) | (0.0794) | (0.1004) |
| A levels |  |  |  | 0.1990*** | 0.2104*** | 0.0987** | 0.0893* | 0.0854* | 0.1327** |
|  |  |  |  | (0.0451) | (0.0413) | (0.0473) | (0.0476) | (0.0484) | (0.0594) |
| HE and TQ |  |  |  | 0.3615*** | 0.3675*** | 0.2017*** | 0.1881*** | 0.1847*** | 0.2437*** |
|  |  |  |  | (0.0483) | (0.0439) | (0.0475) | (0.0468) | (0.0481) | (0.0575) |
| first degree |  |  |  | 0.5134*** | 0.5137*** | 0.3128*** | 0.2859*** | 0.2807*** | 0.3476*** |
|  |  |  |  | (0.0485) | (0.0441) | (0.0499) | (0.0478) | (0.0491) | (0.0604) |
| higher degree |  |  |  | 0.6842*** | 0.6862*** | 0.4630*** | 0.4288*** | 0.4176*** | 0.4826*** |
|  |  |  |  | (0.0496) | (0.0449) | (0.0510) | (0.0488) | (0.0508) | (0.0595) |
| experience |  |  |  | 0.0238*** | 0.0220*** | 0.0174*** | 0.0163*** | 0.0156*** | 0.0166*** |
|  |  |  |  | (0.0018) | (0.0018) | (0.0017) | (0.0018) | (0.0017) | (0.0022) |
| experience squared | |  |  | -0.0003*** | -0.0003*** | -0.0002*** | -0.0002*** | -0.0002*** | -0.0002*** |
|  |  |  |  | (0.0000) | (0.0000) | (0.0000) | (0.0000) | (0.0000) |  |
| age |  |  |  |  | 0.0003 | 0.0005 | 0.0016*** | 0.0016*** | 0.0015** |
|  |  |  |  |  | (0.0005) | (0.0004) | (0.0004) | (0.0004) | (0.0006) |
| ethnic minority |  |  |  |  | 0.0280 | 0.0180 | 0.0139 | -0.0146 | -0.0230 |
|  |  |  |  |  | (0.0177) | (0.0151) | (0.0152) | (0.0144) | (0.0192) |
| live in couples |  |  |  |  | 0.0494*** | 0.0424*** | 0.0415*** | 0.0474*** |  |
|  |  |  |  |  | (0.0094) | (0.0085) | (0.0081) | (0.0083) |  |
| dependent children |  |  |  |  | 0.0468*** | 0.0367*** | 0.0453*** | 0.0470*** | 0.0576*** |
|  |  |  |  |  | (0.0103) | (0.0091) | (0.0092) | (0.0091) | (0.0105) |
| disability |  |  |  |  | -0.0464*** | -0.0348*** | -0.0299*** | -0.0311*** | -0.0313*** |
|  |  |  |  |  | (0.0113) | (0.0091) | (0.0089) | (0.0087) | (0.0108) |
| carer |  |  |  |  | 0.0016 | 0.0061 | 0.0027 | 0.0031 | 0.0045 |
|  |  |  |  |  | (0.0091) | (0.0090) | (0.0085) | (0.0081) | (0.0112) |
| foreign |  |  |  |  | -0.0203 | -0.0165 | -0.0163 | -0.0295** | -0.0353** |
|  |  |  |  |  | (0.0168) | (0.0150) | (0.0146) | (0.0136) | (0.0166) |
|  | | | | |  |  |  |  |  |
| Occupational group (omitted group: Registered nurse and midwives) | | | | |  |  |  |  |  |
| allied |  |  |  |  |  | 0.0238* | 0.0300** | 0.0324** | 0.0435** |
|  |  |  |  |  |  | (0.0127) | (0.0127) | (0.0125) | (0.0178) |
| ambulance |  |  |  |  |  | 0.0181 | 0.0278 | 0.0438 | 0.0184 |
|  |  |  |  |  |  | (0.0479) | (0.0472) | (0.0632) | (0.0634) |
| public health |  |  |  |  |  | 0.0178 | 0.0229 | 0.0230 | 0.0082 |
|  |  |  |  |  |  | (0.0375) | (0.0378) | (0.0382) | (0.0487) |
| commissioning manager | |  |  |  |  | 0.1526*** | 0.1372*** | 0.1344*** | 0.1692*** |
|  |  |  |  |  |  | (0.0347) | (0.0322) | (0.0318) | (0.0407) |
| nursing auxiliary |  |  |  |  |  | -0.1401*** | -0.1362*** | -0.1343*** | -0.1350*** |
|  |  |  |  |  |  | (0.0226) | (0.0214) | (0.0202) | (0.0288) |
| social care |  |  |  |  |  | 0.1690*** | 0.1526*** | 0.1640*** | 0.1739*** |
|  |  |  |  |  |  | (0.0385) | (0.0399) | (0.0414) | (0.0564) |
| wider |  |  |  |  |  | 0.0762*** | 0.0640*** | 0.0674*** | 0.0762*** |
|  |  |  |  |  |  | (0.0208) | (0.0196) | (0.0193) | (0.0269) |
| general management | |  |  |  |  | 0.4170*** | 0.3747*** | 0.3710*** | 0.3737*** |
|  |  |  |  |  |  | (0.0245) | (0.0239) | (0.0223) | (0.0315) |
| other |  |  |  |  |  | 0.0565*** | 0.0439** | 0.0461** | 0.0611** |
|  |  |  |  |  |  | (0.0201) | (0.0203) | (0.0197) | (0.0269) |
| health professional |  |  |  |  |  | 0.2265*** | 0.2316*** | 0.2288*** | 0.2271*** |
|  |  |  |  |  |  | (0.0179) | (0.0174) | (0.0167) | (0.0225) |
| part time |  |  |  |  |  |  | -0.0874*** | -0.0838*** | -0.0742*** |
|  |  |  |  |  |  |  | (0.0111) | (0.0103) | (0.0122) |
| job permanent |  |  |  |  |  |  | -0.0091 | -0.0057 | -0.0055 |
|  |  |  |  |  |  |  | (0.0173) | (0.0162) | (0.0184) |
| trade union |  |  |  |  |  |  | -0.0319*** | -0.0286*** | -0.0367*** |
|  |  |  |  |  |  |  | (0.0098) | (0.0094) | (0.0130) |
| mentor |  |  |  |  |  |  | -0.0516*** | -0.0464*** | -0.0488*** |
|  |  |  |  |  |  |  | (0.0074) | (0.0078) | (0.0102) |
| happy training |  |  |  |  |  |  | 0.0729*** | 0.0689*** | 0.0739*** |
|  |  |  |  |  |  |  | (0.0100) | (0.0102) | (0.0130) |
| friend |  |  |  |  |  |  | 0.0132 | 0.0102 | 0.0020 |
|  |  |  |  |  |  |  | (0.0082) | (0.0083) | (0.0091) |
| responsive hours |  |  |  |  |  |  | 0.0407*** | 0.0433*** | 0.0402*** |
|  |  |  |  |  |  |  | (0.0096) | (0.0087) | (0.0138) |
| pressure |  |  |  |  |  |  | 0.0310*** | 0.0343*** | 0.0382*** |
|  |  |  |  |  |  |  | (0.0085) | (0.0084) | (0.0108) |
| coworker support |  |  |  |  |  |  | 0.0189* | 0.0184* | 0.0150 |
|  |  |  |  |  |  |  | (0.0098) | (0.0099) | (0.0133) |
| work-life balance |  |  |  |  |  |  | -0.0294*** | -0.0278*** | -0.0325*** |
|  |  |  |  |  |  |  | (0.0091) | (0.0088) | (0.0108) |
| supervisor support |  |  |  |  |  |  | 0.0306*** | 0.0287*** | 0.0290** |
|  |  |  |  |  |  |  | (0.0097) | (0.0097) | (0.0127) |
| cooperative |  |  |  |  |  |  |  | 0.0088 | 0.0102 |
|  |  |  |  |  |  |  |  | (0.0081) | (0.0101) |
| NHS England region (omitted group: North of England) | | | |  |  |  |  |  |  |
| Midlands and East of England | |  |  |  |  |  |  | 0.0252* | 0.0296 |
|  |  |  |  |  |  |  |  | (0.0152) | (0.0180) |
| London |  |  |  |  |  |  |  | 0.1224*** | 0.1403*** |
|  |  |  |  |  |  |  |  | (0.0169) | (0.0216) |
| South West |  |  |  |  |  |  |  | 0.0065 | 0.0058 |
|  |  |  |  |  |  |  |  | (0.0193) | (0.0244) |
|  |  |  |  |  |  |  |  |  |  |
| South East |  |  |  |  |  |  |  | 0.0378** | 0.0478** |
|  |  |  |  |  |  |  |  | (0.0161) | (0.0201) |
| Trust type (omitted group: Acute Trusts) | | |  |  |  |  |  |  |  |
| Acute Specialist Trusts | |  |  |  |  |  |  | 0.0216 | 0.0144 |
|  |  |  |  |  |  |  |  | (0.0306) | (0.0353) |
| Ambulance Trusts | |  |  |  |  |  |  | -0.0051 | 0.0419 |
|  |  |  |  |  |  |  |  | (0.0698) | (0.0608) |
| Combined Acute and Community Trusts | | |  |  |  |  |  | 0.0000 | 0.0092 |
|  |  |  |  |  |  |  |  | (0.0165) | (0.0205) |
| Combined Mental Health / Learning Disability and Community Trusts | | | | |  |  |  | -0.0437** | -0.0321 |
|  |  |  |  |  |  |  |  | (0.0209) | (0.0239) |
| Community Trusts | |  |  |  |  |  |  | -0.0415** | -0.0485*** |
|  |  |  |  |  |  |  |  | (0.0179) | (0.0164) |
| Mental Health / Learning Disability Trusts | | | |  |  |  |  | -0.0084 | -0.0002 |
|  |  |  |  |  |  |  |  | (0.0132) | (0.0165) |
|  |  |  |  |  |  |  |  |  |  |
| constant | 2.7334*** | 2.7334*** | 2.7342*** | 2.0094*** | 1.9695*** | 2.0368*** | 1.9993*** | 1.9776*** | 1.9485*** |
|  | (0.0159) | (0.0165) | (0.0166) | (0.0514) | (0.0511) | (0.0569) | (0.0554) | (0.0561) | (0.0784) |
| Observations | 3556 | 3556 | 3556 | 3556 | 3556 | 3556 | 3556 | 3556 | 2443 |
| R-squared | 0.002 | 0.002 | 0.006 | 0.457 | 0.470 | 0.580 | 0.611 | 0.6241 | 0.6205 |
| Adj. R-squared | 0.002 | 0.001 | 0.005 | 0.455 | 0.467 | 0.577 | 0.607 | 0.6186 | 0.6126 |
| Standard errors are in parentheses (clustered at individual Trust level). * p<0.10, ** p<0.05, *** p<0.01. | | | | | | | | | |
