# Supplementary material for: Pay gaps in the National Health Service: Gender and sexuality
Source: PLoS One. 2026 Mar 4;21(3):e0342384. doi: 10.1371/journal.pone.0342384 (PMC12959664; doi:10.1371/journal.pone.0342384)
Supplement: S4 Table — (DOCX) [file pone.0342384.s004.docx]

| **S4 TABLE. Determinants of log earnings (OLS estimates), men**. | | | | | | | | |
| --- | --- | --- | --- | --- | --- | --- | --- | --- |
| Dependent variable is ln(salary) | (1) | (2) | (3) | (4) | (5) | (6) | (7) | (8) |
|  | Min | Base | +HC | +Demog | +Occup | +Job/Work | Broader | Coupled |
| GB+ | 0.0258 |  |  |  |  |  |  |  |
|  | (0.0281) |  |  |  |  |  |  |  |
| no disclose & GB+ |  | -0.0806** | -0.0666** | -0.0173 | -0.0271 | -0.0235 | -0.0254 | -0.0400 |
|  |  | (0.0360) | (0.0300) | (0.0300) | (0.0312) | (0.0295) | (0.0280) | (0.0390) |
| disclose & GB+ |  | 0.0953*** | 0.0370 | 0.0958*** | 0.0925*** | 0.0830*** | 0.0722*** | 0.0692 |
|  |  | (0.0361) | (0.0286) | (0.0299) | (0.0290) | (0.0294) | (0.0270) | (0.0423) |
|  |  |  |  |  |  |  |  |  |
| Qualifications (omitted group: min qual) | | |  |  |  |  |  |  |
| O level |  |  | -0.0780 | -0.0607 | -0.1015 | -0.1198 | -0.1500 | -0.2223** |
|  |  |  | (0.1314) | (0.1323) | (0.1204) | (0.0910) | (0.0972) | (0.1078) |
| GCSE |  |  | -0.0053 | 0.0259 | -0.0619 | -0.1089 | -0.1460 | -0.1508 |
|  |  |  | (0.1276) | (0.1248) | (0.1220) | (0.0937) | (0.1059) | (0.1266) |
| trade |  |  | 0.1218 | 0.1336 | 0.0400 | 0.0213 | -0.0023 | -0.0139 |
|  |  |  | (0.2183) | (0.2133) | (0.2056) | (0.1712) | (0.1751) | (0.1987) |
| A levels |  |  | 0.0461 | 0.0844 | 0.0105 | -0.0014 | -0.0242 | -0.0662 |
|  |  |  | (0.0994) | (0.0985) | (0.1064) | (0.0773) | (0.0871) | (0.1006) |
| HE and TQ |  |  | 0.2514** | 0.2780** | 0.1460 | 0.1059 | 0.0726 | 0.0809 |
|  |  |  | (0.1202) | (0.1194) | (0.1214) | (0.0856) | (0.0962) | (0.0996) |
| first degree |  |  | 0.3802*** | 0.3983*** | 0.2402* | 0.2036** | 0.1726* | 0.1561 |
|  |  |  | (0.1275) | (0.1225) | (0.1261) | (0.0914) | (0.1008) | (0.1087) |
| higher degree |  |  | 0.5323*** | 0.5543*** | 0.3539*** | 0.3098*** | 0.2745*** | 0.2624*** |
|  |  |  | (0.1144) | (0.1088) | (0.1179) | (0.0823) | (0.0948) | (0.1002) |
| experience |  |  | 0.0265*** | 0.0212*** | 0.0202*** | 0.0178*** | 0.0170*** | 0.0190*** |
|  |  |  | (0.0037) | (0.0039) | (0.0036) | (0.0036) | (0.0034) | (0.0038) |
| experience squared | |  | -0.0004*** | -0.0003*** | -0.0003*** | -0.0003*** | -0.0003*** | -0.0003*** |
|  |  |  | (0.0001) | (0.0001) | (0.0001) | (0.0001) | (0.0001) | (0.0001) |
| age |  |  |  | 0.0023* | 0.0015 | 0.0027** | 0.0031*** | 0.0034** |
|  |  |  |  | (0.0013) | (0.0012) | (0.0012) | (0.0011) | (0.0013) |
| ethnic minority |  |  |  | 0.0387 | 0.0193 | 0.0214 | -0.0042 | 0.0112 |
|  |  |  |  | (0.0353) | (0.0322) | (0.0308) | (0.0333) | (0.0434) |
| live in couples |  |  |  | 0.0889*** | 0.0832*** | 0.0685*** | 0.0681*** |  |
|  |  |  |  | (0.0204) | (0.0190) | (0.0198) | (0.0181) |  |
| dependent children |  |  |  | 0.0887*** | 0.0691*** | 0.0591*** | 0.0722*** | 0.0698** |
|  |  |  |  | (0.0251) | (0.0227) | (0.0220) | (0.0216) | (0.0270) |
| disability |  |  |  | -0.0397 | -0.0195 | -0.0217 | -0.0228 | -0.0119 |
|  |  |  |  | (0.0253) | (0.0214) | (0.0205) | (0.0210) | (0.0259) |
| carer |  |  |  | 0.0050 | 0.0097 | 0.0157 | 0.0123 | 0.0199 |
|  |  |  |  | (0.0262) | (0.0209) | (0.0208) | (0.0190) | (0.0237) |
| foreign |  |  |  | -0.0635** | -0.0326 | -0.0414 | -0.0517* | -0.0598 |
|  |  |  |  | (0.0319) | (0.0299) | (0.0302) | (0.0311) | (0.0393) |
|  |  |  |  |  |  |  |  |  |
| Occupational group (omitted group: Registered nurse and midwives) | | | | |  |  |  |  |
| allied health |  |  |  |  | 0.0524* | 0.0553* | 0.0546* | 0.0794* |
|  |  |  |  |  | (0.0298) | (0.0308) | (0.0307) | (0.0409) |
| Ambulance (op) |  |  |  |  | 0.0569 | 0.0667 | 0.2914*** | 0.3320*** |
|  |  |  |  |  | (0.0537) | (0.0505) | (0.0510) | (0.0663) |
| public health |  |  |  |  | -0.0063 | 0.0059 | -0.0243 | -0.0925 |
|  |  |  |  |  | (0.0783) | (0.0792) | (0.0950) | (0.0927) |
| commissioning manager | |  |  |  | 0.1168* | 0.1100** | 0.1210** | 0.1495** |
|  |  |  |  |  | (0.0611) | (0.0535) | (0.0532) | (0.0704) |
| nursing auxiliary |  |  |  |  | -0.1880*** | -0.2005*** | -0.1876*** | -0.1547*** |
|  |  |  |  |  | (0.0482) | (0.0469) | (0.0470) | (0.0536) |
| social care |  |  |  |  | 0.4024*** | 0.3301** | 0.3991*** | 0.4246*** |
|  |  |  |  |  | (0.1471) | (0.1544) | (0.1405) | (0.1493) |
| wider health |  |  |  |  | 0.1022** | 0.0915* | 0.0972** | 0.1669** |
|  |  |  |  |  | (0.0484) | (0.0468) | (0.0439) | (0.0643) |
| general management | |  |  |  | 0.3639*** | 0.3268*** | 0.3400*** | 0.3446*** |
|  |  |  |  |  | (0.0615) | (0.0587) | (0.0556) | (0.0691) |
| other |  |  |  |  | 0.0477 | 0.0369 | 0.0316 | 0.0737 |
|  |  |  |  |  | (0.0502) | (0.0493) | (0.0472) | (0.0572) |
| health professional |  |  |  |  | 0.1842*** | 0.1775*** | 0.1868*** | 0.1795*** |
|  |  |  |  |  | (0.0426) | (0.0428) | (0.0411) | (0.0547) |
| part time |  |  |  |  |  | -0.0858** | -0.0792** | -0.0051 |
|  |  |  |  |  |  | (0.0336) | (0.0318) | (0.0398) |
| job permanent |  |  |  |  |  | 0.0426 | 0.0503* | 0.1327*** |
|  |  |  |  |  |  | (0.0302) | (0.0288) | (0.0489) |
| trade union |  |  |  |  |  | -0.0183 | -0.0139 | -0.0273 |
|  |  |  |  |  |  | (0.0237) | (0.0242) | (0.0290) |
| mentor |  |  |  |  |  | -0.0495** | -0.0425* | -0.0375 |
|  |  |  |  |  |  | (0.0238) | (0.0236) | (0.0304) |
| happy training |  |  |  |  |  | 0.0680*** | 0.0677*** | 0.0707*** |
|  |  |  |  |  |  | (0.0216) | (0.0211) | (0.0257) |
| friend |  |  |  |  |  | 0.0544*** | 0.0453** | 0.0324 |
|  |  |  |  |  |  | (0.0196) | (0.0200) | (0.0231) |
| responsive hours |  |  |  |  |  | 0.0259 | 0.0334 | 0.0166 |
|  |  |  |  |  |  | (0.0233) | (0.0230) | (0.0323) |
| pressure |  |  |  |  |  | 0.0644*** | 0.0646*** | 0.0848*** |
|  |  |  |  |  |  | (0.0214) | (0.0206) | (0.0282) |
| coworker support |  |  |  |  |  | 0.0405 | 0.0366 | 0.0321 |
|  |  |  |  |  |  | (0.0254) | (0.0253) | (0.0358) |
| work-life balance |  |  |  |  |  | -0.0319 | -0.0199 | -0.0151 |
|  |  |  |  |  |  | (0.0239) | (0.0238) | (0.0264) |
| supervisor support |  |  |  |  |  | 0.0539** | 0.0555** | 0.0792*** |
|  |  |  |  |  |  | (0.0245) | (0.0243) | (0.0296) |
| cooperative |  |  |  |  |  |  | -0.0009 | -0.0017 |
|  |  |  |  |  |  |  | (0.0221) | (0.0279) |
| NHS England region (omitted group: North of England) | | | |  |  |  |  |  |
| Midlands and East of England | |  |  |  |  |  | 0.0470 | 0.0400 |
|  |  |  |  |  |  |  | (0.0304) | (0.0374) |
| London |  |  |  |  |  |  | 0.1758*** | 0.1847*** |
|  |  |  |  |  |  |  | (0.0325) | (0.0427) |
| South West |  |  |  |  |  |  | 0.0497 | 0.0596 |
|  |  |  |  |  |  |  | (0.0376) | (0.0434) |
| South East |  |  |  |  |  |  | 0.0524 | 0.0660* |
|  |  |  |  |  |  |  | (0.0317) | (0.0394) |
|  |  |  |  |  |  |  |  |  |
| Trust type (omitted group: Acute Trusts) | | |  |  |  |  |  |  |
| Acute Specialist Trusts | |  |  |  |  |  | -0.0441 | -0.0496 |
|  |  |  |  |  |  |  | (0.0511) | (0.0730) |
| Ambulance Trusts | |  |  |  |  |  | -0.2429*** | -0.2298*** |
|  |  |  |  |  |  |  | (0.0471) | (0.0715) |
| Combined Acute and Community Trusts | | |  |  |  |  | 0.0079 | 0.0317 |
|  |  |  |  |  |  |  | (0.0334) | (0.0411) |
| Combined Mental Health / Learning Disability and Community Trusts | | | | |  |  | -0.1338*** | -0.1436*** |
|  |  |  |  |  |  |  | (0.0341) | (0.0446) |
| Community Trusts | |  |  |  |  |  | 0.0043 | -0.0010 |
|  |  |  |  |  |  |  | (0.0293) | (0.0374) |
| Mental Health / Learning Disability Trusts | | |  |  |  |  | -0.0267 | 0.0071 |
|  |  |  |  |  |  |  | (0.0258) | (0.0345) |
|  |  |  |  |  |  |  |  |  |
| constant | 2.7702*** | 2.7702*** | 2.1999*** | 2.0399*** | 2.0798*** | 1.9633*** | 1.9228*** | 1.8400*** |
|  | (0.0205) | (0.0205) | (0.1175) | (0.1140) | (0.1178) | (0.0939) | (0.0992) | (0.1144) |
| Observations | 753 | 753 | 753 | 753 | 753 | 753 | 753 | 524 |
| R-squared | 0.0009 | 0.0148 | 0.4031 | 0.4364 | 0.5386 | 0.5732 | 0.6018 | 0.5786 |
| Adj. R-squared | -0.0004 | 0.0122 | 0.3943 | 0.4226 | 0.5208 | 0.5499 | 0.5734 | 0.5350 |
| Standard errors are in parentheses (clustered at individual Trust level). * p<0.10, ** p<0.05, *** p<0.01. | | | | | | | | |
