# Supplementary material for: Pay gaps in the National Health Service: Gender and sexuality
Source: PLoS One. 2026 Mar 4;21(3):e0342384. doi: 10.1371/journal.pone.0342384 (PMC12959664; doi:10.1371/journal.pone.0342384)
Supplement: S5 Table — (DOCX) [file pone.0342384.s005.docx]

| **S5 TABLE. Determinants of log earnings (OLS estimates), women**. | | | | | | | | |
| --- | --- | --- | --- | --- | --- | --- | --- | --- |
|  | (1) | (2) | (3) | (4) | (5) | (6) | (7) | (8) |
|  | Min | Base | +HC | +Demog | +Occup | +Job/Work | Broader | Coupled |
| LB+ | -0.0187 |  |  |  |  |  |  |  |
|  | (0.0255) |  |  |  |  |  |  |  |
| no disclose & LB+ |  | -0.0477 | -0.0473** | -0.0291 | -0.0282 | -0.0395** | -0.0500** | -0.0439 |
|  |  | (0.0291) | (0.0234) | (0.0221) | (0.0184) | (0.0178) | (0.0194) | (0.0273) |
| disclose & LB+ |  | 0.0210 | 0.0274 | 0.0406 | 0.0561** | 0.0489** | 0.0394* | 0.0323 |
|  |  | (0.0377) | (0.0261) | (0.0250) | (0.0238) | (0.0241) | (0.0232) | (0.0312) |
|  |  |  |  |  |  |  |  |  |
| Qualifications (omitted group: min quals) | | |  |  |  |  |  |  |
| O level |  |  | 0.1116*** | 0.1167*** | 0.0347 | 0.0358 | 0.0335 | 0.1201*** |
|  |  |  | (0.0313) | (0.0309) | (0.0404) | (0.0390) | (0.0399) | (0.0353) |
| GCSE |  |  | 0.1812*** | 0.1767*** | 0.0657 | 0.0677 | 0.0619 | 0.1504*** |
|  |  |  | (0.0350) | (0.0341) | (0.0414) | (0.0414) | (0.0415) | (0.0388) |
| trade |  |  | 0.2039*** | 0.1837*** | 0.1020* | 0.0786 | 0.0747 | 0.1784*** |
|  |  |  | (0.0701) | (0.0599) | (0.0578) | (0.0543) | (0.0501) | (0.0523) |
| A levels |  |  | 0.2382*** | 0.2361*** | 0.1192*** | 0.1136*** | 0.1105*** | 0.1890*** |
|  |  |  | (0.0329) | (0.0338) | (0.0403) | (0.0400) | (0.0398) | (0.0334) |
| HE and TQ |  |  | 0.3879*** | 0.3808*** | 0.2102*** | 0.2041*** | 0.2014*** | 0.2810*** |
|  |  |  | (0.0337) | (0.0349) | (0.0416) | (0.0414) | (0.0422) | (0.0361) |
| first degree |  |  | 0.5465*** | 0.5340*** | 0.3249*** | 0.3032*** | 0.2975*** | 0.3911*** |
|  |  |  | (0.0300) | (0.0308) | (0.0412) | (0.0396) | (0.0400) | (0.0344) |
| higher degree |  |  | 0.7224*** | 0.7110*** | 0.4863*** | 0.4557*** | 0.4451*** | 0.5386*** |
|  |  |  | (0.0322) | (0.0322) | (0.0422) | (0.0411) | (0.0424) | (0.0359) |
| experience |  |  | 0.0232*** | 0.0225*** | 0.0169*** | 0.0162*** | 0.0158*** | 0.0165*** |
|  |  |  | (0.0018) | (0.0018) | (0.0016) | (0.0018) | (0.0017) | (0.0022) |
| experience squared | |  | -0.0003*** | -0.0003*** | -0.0002*** | -0.0002*** | -0.0002*** | -0.0002*** |
|  |  |  | (0.0000) | (0.0000) | (0.0000) | (0.0000) | (0.0000) | (0.0000) |
| age |  |  |  | -0.0006 | 0.0001 | 0.0012** | 0.0012** | 0.0012* |
|  |  |  |  | (0.0007) | (0.0005) | (0.0005) | (0.0005) | (0.0007) |
| ethnic minority |  |  |  | 0.0236 | 0.0145 | 0.0077 | -0.0238 | -0.0324 |
|  |  |  |  | (0.0226) | (0.0181) | (0.0175) | (0.0165) | (0.0230) |
| live in couples |  |  |  | 0.0383*** | 0.0322*** | 0.0341*** | 0.0389*** |  |
|  |  |  |  | (0.0105) | (0.0096) | (0.0090) | (0.0092) |  |
| dependent children |  |  |  | 0.0368*** | 0.0306*** | 0.0430*** | 0.0435*** | 0.0611*** |
|  |  |  |  | (0.0114) | (0.0100) | (0.0100) | (0.0097) | (0.0108) |
| disability |  |  |  | -0.0485*** | -0.0372*** | -0.0306*** | -0.0323*** | -0.0338*** |
|  |  |  |  | (0.0118) | (0.0099) | (0.0097) | (0.0094) | (0.0115) |
| carer |  |  |  | 0.0032 | 0.0066 | 0.0010 | 0.0010 | 0.0035 |
|  |  |  |  | (0.0104) | (0.0095) | (0.0086) | (0.0083) | (0.0118) |
| foreign |  |  |  | -0.0063 | -0.0101 | -0.0097 | -0.0218 | -0.0267 |
|  |  |  |  | (0.0182) | (0.0160) | (0.0156) | (0.0151) | (0.0185) |
|  |  |  |  |  |  |  |  |  |
| Occupational group (omitted group: Registered nurse and midwives) | | | | |  |  |  |  |
| allied |  |  |  |  | 0.0227 | 0.0275** | 0.0291** | 0.0377** |
|  |  |  |  |  | (0.0144) | (0.0136) | (0.0136) | (0.0189) |
| ambulance |  |  |  |  | -0.0575 | -0.0665 | -0.1601*** | -0.1768*** |
|  |  |  |  |  | (0.0607) | (0.0584) | (0.0594) | (0.0670) |
| public health |  |  |  |  | 0.0280 | 0.0326 | 0.0426 | 0.0400 |
|  |  |  |  |  | (0.0488) | (0.0459) | (0.0430) | (0.0523) |
| commissioning manager | |  |  |  | 0.1823*** | 0.1617*** | 0.1553*** | 0.1994*** |
|  |  |  |  |  | (0.0364) | (0.0355) | (0.0352) | (0.0466) |
| nursing auxiliary |  |  |  |  | -0.1145*** | -0.1087*** | -0.1090*** | -0.1073*** |
|  |  |  |  |  | (0.0263) | (0.0249) | (0.0241) | (0.0355) |
| social care |  |  |  |  | 0.1026** | 0.0997** | 0.0972** | 0.0794** |
|  |  |  |  |  | (0.0440) | (0.0432) | (0.0478) | (0.0402) |
| wider |  |  |  |  | 0.0881*** | 0.0733*** | 0.0742*** | 0.0762*** |
|  |  |  |  |  | (0.0224) | (0.0206) | (0.0207) | (0.0287) |
| general management | |  |  |  | 0.4538*** | 0.4082*** | 0.3972*** | 0.4097*** |
|  |  |  |  |  | (0.0252) | (0.0239) | (0.0227) | (0.0326) |
| other |  |  |  |  | 0.0729*** | 0.0577** | 0.0584** | 0.0695** |
|  |  |  |  |  | (0.0232) | (0.0224) | (0.0225) | (0.0323) |
| health professional |  |  |  |  | 0.2506*** | 0.2602*** | 0.2556*** | 0.2615*** |
|  |  |  |  |  | (0.0204) | (0.0198) | (0.0197) | (0.0268) |
| part time |  |  |  |  |  | -0.0856*** | -0.0815*** | -0.0776*** |
|  |  |  |  |  |  | (0.0110) | (0.0106) | (0.0122) |
| job permanent |  |  |  |  |  | -0.0227 | -0.0204 | -0.0183 |
|  |  |  |  |  |  | (0.0201) | (0.0191) | (0.0204) |
| trade union |  |  |  |  |  | -0.0379*** | -0.0350*** | -0.0430*** |
|  |  |  |  |  |  | (0.0104) | (0.0099) | (0.0136) |
| mentor |  |  |  |  |  | -0.0531*** | -0.0485*** | -0.0515*** |
|  |  |  |  |  |  | (0.0079) | (0.0078) | (0.0123) |
| happy training |  |  |  |  |  | 0.0728*** | 0.0677*** | 0.0718*** |
|  |  |  |  |  |  | (0.0105) | (0.0108) | (0.0126) |
| friend |  |  |  |  |  | 0.0036 | 0.0014 | -0.0032 |
|  |  |  |  |  |  | (0.0091) | (0.0092) | (0.0110) |
| responsive hours |  |  |  |  |  | 0.0440*** | 0.0452*** | 0.0425*** |
|  |  |  |  |  |  | (0.0093) | (0.0087) | (0.0111) |
| pressure |  |  |  |  |  | 0.0222** | 0.0255*** | 0.0268** |
|  |  |  |  |  |  | (0.0094) | (0.0095) | (0.0112) |
| coworker support |  |  |  |  |  | 0.0122 | 0.0116 | 0.0047 |
|  |  |  |  |  |  | (0.0128) | (0.0130) | (0.0167) |
| work-life balance |  |  |  |  |  | -0.0288*** | -0.0274*** | -0.0321** |
|  |  |  |  |  |  | (0.0092) | (0.0089) | (0.0125) |
| supervisor support |  |  |  |  |  | 0.0259** | 0.0242** | 0.0197 |
|  |  |  |  |  |  | (0.0107) | (0.0100) | (0.0130) |
| cooperative |  |  |  |  |  |  | 0.0110 | 0.0144 |
|  |  |  |  |  |  |  | (0.0088) | (0.0107) |
|  |  |  |  |  |  |  |  |  |
| NHS England region (omitted group: North of England) | | | |  |  |  |  |  |
| Midlands and East of England | |  |  |  |  |  | 0.0210 | 0.0248 |
|  |  |  |  |  |  |  | (0.0165) | (0.0194) |
| London |  |  |  |  |  |  | 0.1067*** | 0.1219*** |
|  |  |  |  |  |  |  | (0.0174) | (0.0206) |
| South West |  |  |  |  |  |  | -0.0031 | -0.0110 |
|  |  |  |  |  |  |  | (0.0197) | (0.0231) |
| South East |  |  |  |  |  |  | 0.0362** | 0.0421** |
|  |  |  |  |  |  |  | (0.0164) | (0.0206) |
| Trust type (omitted group: Acute Trusts) | | |  |  |  |  |  |  |
| Acute Specialist Trusts | |  |  |  |  |  | 0.0498 | 0.0423 |
|  |  |  |  |  |  |  | (0.0341) | (0.0361) |
| Ambulance Trusts | |  |  |  |  |  | 0.1120* | 0.1331* |
|  |  |  |  |  |  |  | (0.0655) | (0.0705) |
| Combined Acute and Community Trusts | | |  |  |  |  | 0.0034 | 0.0102 |
|  |  |  |  |  |  |  | (0.0168) | (0.0207) |
| Combined Mental Health / Learning Disability and Community Trusts | | | | |  |  | -0.0198 | 0.0011 |
|  |  |  |  |  |  |  | (0.0238) | (0.0257) |
| Community Trusts | |  |  |  |  |  | -0.0468** | -0.0523*** |
|  |  |  |  |  |  |  | (0.0180) | (0.0187) |
| Mental Health / Learning Disability Trusts | | |  |  |  |  | -0.0021 | 0.0048 |
|  |  |  |  |  |  |  | (0.0157) | (0.0190) |
|  |  |  |  |  |  |  |  |  |
| constant | 2.7349*** | 2.7349*** | 1.9777*** | 1.9855*** | 2.0290*** | 2.0128*** | 1.9988*** | 1.9336*** |
|  | (0.0169) | (0.0169) | (0.0335) | (0.0476) | (0.0532) | (0.0564) | (0.0579) | (0.0658) |
| Observations | 2803 | 2803 | 2803 | 2803 | 2803 | 2803 | 2803 | 1919 |
| R-squared | 0.0002 | 0.0010 | 0.4739 | 0.4851 | 0.6005 | 0.6319 | 0.6431 | 0.6508 |
| Adj. R-squared | -0.0001 | 0.0002 | 0.4718 | 0.4818 | 0.5965 | 0.6267 | 0.6367 | 0.6416 |
| Standard errors are in parentheses (clustered at individual Trust level). * p<0.10, ** p<0.05, *** p<0.01. | | | | | | | | |
