# Supplementary material for: Pay gaps in the National Health Service: Gender and sexuality
Source: PLoS One. 2026 Mar 4;21(3):e0342384. doi: 10.1371/journal.pone.0342384 (PMC12959664; doi:10.1371/journal.pone.0342384)
Supplement: S6 Table — (DOCX) [file pone.0342384.s006.docx]

| **TABLE S6. Determinants of log earnings, decomposition (OLS estimates).** | | | |
| --- | --- | --- | --- |
| Dependent variable is ln(salary) | (1) | (2) | (3) |
|  | Total | Men | Women |
| men | 0.038*** |  |  |
|  | (0.010) |  |  |
| LGB+ |  |  |  |
| no disclose & LGB+ | -0.049*** | -0.025 | -0.050** |
|  | (0.016) | (0.028) | (0.019) |
| disclose & LGB+ | 0.043** | 0.072*** | 0.039* |
|  | (0.017) | (0.027) | (0.023) |
|  |  |  |  |
| Qualifications (omitted group: min qual) |  |  |  |
| O level | 0.000 | ***-0.150*** | ***0.033*** |
|  | (0.051) | (0.097) | (0.039) |
| GCSE | 0.027 | ***-0.146*** | ***0.061*** |
|  | (0.053) | (0.105) | (0.041) |
| trade | 0.093 | -0.002 | 0.074 |
|  | (0.079) | (0.175) | (0.050) |
| A levels | 0.085* | -0.024 | 0.110*** |
|  | (0.048) | (0.087) | (0.039) |
| HE and TQ | 0.184*** | 0.072 | 0.201*** |
|  | (0.048) | (0.096) | (0.042) |
| first degree | 0.280*** | 0.172* | 0.297*** |
|  | (0.049) | (0.100) | (0.040) |
| higher degree | 0.417*** | **0.274***** | **0.445***** |
|  | (0.050) | (0.094) | (0.042) |
| experience | 0.015*** | 0.017*** | 0.015*** |
|  | (0.001) | (0.003) | (0.001) |
| experience squared | -0.000*** | -0.000*** | -0.000*** |
|  | (0.000) | (0.000) | (0.000) |
| age | 0.001*** | 0.003*** | 0.001** |
|  | (0.001) | (0.001) | (0.000) |
| ethnic minority | -0.014 | -0.004 | -0.023 |
|  | (0.014) | (0.033) | (0.016) |
| live in couples | 0.047*** | 0.068*** | 0.038*** |
|  | (0.008) | (0.018) | (0.009) |
| dependent children | 0.047*** | 0.072*** | 0.043*** |
|  | (0.009) | (0.021) | (0.009) |
| disability | -0.031*** | -0.022 | -0.032*** |
|  | (0.008) | (0.021) | (0.009) |
| carer | 0.003 | 0.012 | 0.001 |
|  | (0.008) | (0.019) | (0.008) |
| foreign | -0.029** | -0.051* | -0.021 |
|  | (0.013) | (0.031) | (0.015) |
|  |  |  |  |
| Occupational group (omitted group: Registered nurse and midwives) |  |  |  |
| allied | 0.032** | 0.054* | 0.029** |
|  | (0.012) | (0.030) | (0.013) |
| ambulance | 0.043 | ***0.291****** | ***-0.160****** |
|  | (0.063) | (0.051) | (0.059) |
| public health | 0.023 | -0.024 | 0.042 |
|  | (0.038) | (0.095) | (0.043) |
| commissioning manager | 0.134*** | 0.121** | 0.155*** |
|  | (0.031) | (0.053) | (0.035) |
| nursing auxiliary | -0.134*** | -0.187*** | -0.109*** |
|  | (0.020) | (0.047) | (0.024) |
| social care | 0.164*** | **0.399***** | **0.097**** |
|  | (0.041) | (0.140) | (0.047) |
| wider | 0.067*** | 0.097** | 0.074*** |
|  | (0.019) | (0.043) | (0.020) |
| general management | 0.371*** | 0.340*** | 0.397*** |
|  | (0.022) | (0.055) | (0.022) |
| other | 0.046** | 0.031 | 0.058** |
|  | (0.019) | (0.047) | (0.022) |
| health professional | 0.228*** | 0.186*** | 0.255*** |
|  | (0.016) | (0.041) | (0.019) |
| part time | -0.083*** | -0.079** | -0.081*** |
|  | (0.010) | (0.031) | (0.010) |
| job permanent | -0.005 | ***0.050**** | ***-0.020*** |
|  | (0.016) | (0.028) | (0.019) |
| trade union | -0.028*** | -0.013 | -0.035*** |
|  | (0.009) | (0.024) | (0.009) |
| mentor | -0.046*** | -0.042* | -0.048*** |
|  | (0.007) | (0.023) | (0.007) |
| happy training | 0.068*** | 0.067*** | 0.067*** |
|  | (0.010) | (0.021) | (0.010) |
| friend | 0.010 | ***0.045***** | ***0.001*** |
|  | (0.008) | (0.020) | (0.009) |
| responsive hours | 0.043*** | 0.033 | 0.045*** |
|  | (0.008) | (0.023) | (0.008) |
| pressure | 0.034*** | **0.064***** | **0.025***** |
|  | (0.008) | (0.020) | (0.009) |
| coworker support | 0.018* | 0.036 | 0.011 |
|  | (0.009) | (0.025) | (0.013) |
| work-life balance | -0.027*** | -0.019 | -0.027*** |
|  | (0.008) | (0.023) | (0.008) |
| supervisor support | 0.028*** | 0.055** | 0.024** |
|  | (0.009) | (0.024) | (0.010) |
|  |  |  |  |
| NHS England region (omitted group: North of England) |  |  |  |
| Midlands and East of England | 0.025* | 0.047 | 0.021 |
|  | (0.015) | (0.030) | (0.016) |
| London | 0.122*** | ***0.175****** | ***0.106****** |
|  | (0.016) | (0.032) | (0.017) |
| South West | 0.006 | 0.049 | -0.003 |
|  | (0.019) | (0.037) | (0.019) |
| South East | 0.037** | 0.052 | 0.036** |
|  | (0.016) | (0.031) | (0.016) |
|  |  |  |  |
| Trust type (omitted group: Acute Trusts) |  |  |  |
| Acute Specialist Trusts | 0.021 | **-0.044** | **0.049** |
|  | (0.030) | (0.051) | (0.034) |
| Ambulance Trusts | -0.005 | ***-0.242****** | ***0.112**** |
|  | (0.069) | (0.047) | (0.065) |
|  |  |  |  |
| Combined Acute and Community Trusts | 0.000 | 0.007 | 0.003 |
|  | (0.016) | (0.033) | (0.016) |
| Combined Mental Health / Learning Disability and Community Trusts | -0.043** | ***-0.133****** | ***-0.019*** |
|  | (0.020) | (0.034) | (0.023) |
| Community Trusts | -0.041** | **0.004** | **-0.046**** |
|  | (0.017) | (0.029) | (0.018) |
| Mental Health / Learning Disability Trusts | -0.008 | -0.026 | -0.002 |
|  | (0.013) | (0.025) | (0.015) |
| cooperative | 0.008 | -0.000 | 0.011 |
|  | (0.008) | (0.022) | (0.008) |
| constant | 1.977*** | 1.922*** | 1.998*** |
|  | (0.056) | (0.099) | (0.057) |
|  |  |  |  |
| Observations | 3556 | 753 | 2803 |
| R-squared | 0.624 | 0.601 | 0.643 |
| Adj. R-squared | 0.618 | 0.573 | 0.636 |
| Standard errors are in parentheses (clustered at individual Trust level). * p<0.10, ** p<0.05, *** p<0.01.  Coefficient pairs difference (Men Vs. Women): bold p<0.10, bold and italic p<0.05. | | | |
