# Supplementary material for: Pay gaps in the National Health Service: Gender and sexuality
Source: PLoS One. 2026 Mar 4;21(3):e0342384. doi: 10.1371/journal.pone.0342384 (PMC12959664; doi:10.1371/journal.pone.0342384)
Supplement: S7 Table — (DOCX) [file pone.0342384.s007.docx]

| **S7 TABLE. Broader model comparisons, (OLS estimates).** | | | | |  |
| --- | --- | --- | --- | --- | --- |
| Dependent variable is ln(salary) | **LGB+** | **cisgenderLGB+** | **LG** | **Bisexual** | |
|  | (1) | (2) | (3) | (4) | |
|  |  |  |  |  | |
| **(a) Total sample** |  |  |  |  | |
| man | 0.0389*** | 0.038*** | 0.039*** | 0.038*** | |
|  | (0.011) | (0.010) | (0.011) | (0.012) | |
|  |  |  |  |  | |
| no disclose & sexual identity | -0.049*** | -0.054*** | -0.056** | -0.054** | |
|  | (0.017) | (0.016) | (0.023) | (0.024) | |
| disclose & sexual identity | 0.044** | 0.045** | 0.030* | 0.108** | |
|  | (0.017) | (0.017) | (0.018) | (0.051) | |
| Adj. R-squared | 0.618 | 0.620 | 0.622 | 0.621 | |
| Number observations | 3556 | 3539 | 3404 | 3218 | |
|  |  |  |  |  | |
|  |  |  |  |  | |
| **(b) Men sample** |  |  |  |  | |
|  |  |  |  |  | |
| no disclose & sexual identity | -0.025 | -0.036 | -0.042 | -0.031 | |
|  | (0.028) | (0.026) | (0.032) | (0.047) | |
| disclose & sexual identity | 0.072*** | 0.074*** | 0.067** | 0.140 | |
|  | (0.027) | (0.027) | (0.026) | (0.210) | |
| Adj. R-squared | 0.573 | 0.577 | 0.576 | 0.567 | |
| Number observations | 753 | 747 | 720 | 567 | |
|  |  |  |  |  | |
|  |  |  |  |  | |
| **(c) Women sample** |  |  |  |  | |
|  |  |  |  |  | |
| no disclose & sexual identity | -0.050** | -0.051*** | -0.031 | -0.064** | |
|  | (0.019) | (0.019) | (0.035) | (0.024) | |
| disclose & sexual identity | 0.039* | 0.039* | 0.015 | 0.096** | |
|  | (0.023) | (0.023) | (0.029) | (0.043) | |
| Adj. R-squared | 0.637 | 0.637 | 0.6395 | 0.639 | |
| Number observations | 2803 | 2786 | 2695 | 2651 | |
| Standard errors in parentheses (clustered at individual Trust level). * p<0.10, ** p<0.05, *** p<0.01. No disclose & sexual identity, and disclose & sexual identity, are measured relative to the omitted heterosexual category. In addition to the coefficients listed, the models include additional explanatory and control variables as shown in the broader results provided in Supplementary Appendix Tables: S3 Table, column 8, for the total sample; S4 Table, column 8, for men; and S5 Table, column 8, for women. Results for the transgender and bisexual samples are available upon request. | | | | |  |
